# Randomised Trial Support for Orthopaedic Surgical Procedures

**DOI:** 10.1371/journal.pone.0096745

**Published:** 2014-06-13

**Authors:** Hyeung C. Lim, Sam Adie, Justine M. Naylor, Ian A. Harris

**Affiliations:** 1 Whitlam Orthopaedic Research Centre, Ingham Institute for Applied Medical Research, Liverpool, Australia; 2 University of New South Wales, South Western Sydney Clinical School, Liverpool Hospital, Liverpool, Australia; 3 South Western Sydney Local Health District, Liverpool Hospital, Liverpool, Australia; Georgia Regents University, College of Dental Medicine, United States of America

## Abstract

We investigated the proportion of orthopaedic procedures supported by evidence from randomised controlled trials comparing operative procedures to a non-operative alternative. Orthopaedic procedures conducted in 2009, 2010 and 2011 across three metropolitan teaching hospitals were identified, grouped and ranked according to frequency. Searches of the Cochrane Central Register of Controlled Trials (CENTRAL), the Cochrane Database of Systematic Reviews (CDSR) and the Database of Abstracts of Reviews of Effects (DARE) were performed to identify RCTs evaluating the most commonly performed orthopaedic procedures. Included studies were categorised as “supportive” or “not supportive” of operative treatment. A risk of bias analysis was conducted for included studies using the Cochrane Collaboration's Risk of Bias tool. A total of 9,392 orthopaedic procedures were performed across the index period. 94.6% (8886 procedures) of the total volume, representing the 32 most common operative procedure categories, were used for this analysis. Of the 83 included RCTs, 22.9% (19/83) were classified as supportive of operative intervention. 36.9% (3279/8886) of the total volume of procedures performed were supported by at least one RCT showing surgery to be superior to a non-operative alternative. 19.6% (1743/8886) of the total volume of procedures performed were supported by at least one *low risk of bias RCT* showing surgery to be superior to a non-operative alternative. The level of RCT support for common orthopaedic procedures compares unfavourably with other fields of medicine.

## Introduction

“Evidence based medicine (EBM) is the conscientious, explicit, and judicious use of current best evidence in making decisions about the care of individual patients”[1]. Since this definition by Sackett in 1996, EBM has been adopted and included in most developed medical and health care curricula around the world [2], [3]. The study design that provides the best (least biased) evidence for the efficacy and safety of an intervention is the randomised controlled trial (RCT). The process of randomised allocation equally distributes prognostic factors among study groups, and is therefore the best tool in dealing with confounding [4] Empirical research has shown that healthcare decisions on medical wards are more likely to be based on randomised trial evidence (53%) [5] than those on surgical wards (24% to 26%) [6]–[8].

RCTs assessing operative interventions face particular challenges. Operative procedures are often difficult to standardise [9], [10], are frequently conducted in an emergency setting [11], and patients may find difficulty in accepting either the validity of the non-operative comparison [12] or the risk of potential, and often irreversible, adverse outcomes associated with the operative option [13]. Furthermore, academic surgical units are less common when compared with other specialties [14], and surgeon equipoise, particularly when comparing operative and non-operative treatments, is difficult to obtain [11].

Almost two decades ago, surgical research was ridiculed as a “comic opera” as most peer reviewed publications were found to be case series or professional opinion [15]. While as a proportion of publications surgical RCTs are becoming more common [16]–[18], RCTs comparing operative procedures to (best) non-operative management remain uncommon. These comparisons are important to establish the utility of any operative procedure.

While empirical research has been conducted on the quantity and quality of orthopaedic and surgical randomised trials [7], [19], [20], little is known about whether current clinical orthopaedic practice has an evidence base from randomised trials. An appreciation of what volume and proportion of commonly performed procedures have *any* evidence from randomised trials (when compared to non-operative interventions) is relevant when deciding whether resources are being used appropriately.

Our aims were: i) to determine the proportion of the total number of commonly performed orthopaedic procedures that are supported by RCT evidence (indicating operative treatment may be superior to non-operative treatment), and ii) to investigate the risk of bias in RCTs that have compared commonly performed orthopaedic procedures to non-operative alternatives. A secondary aim was to establish what proportion of procedure *types* was supported by RCT evidence in favour of the operative approach over a non-operative approach.

## Methods

### Identification and Selection of Orthopaedic Procedures

We reviewed the RCT evidence for commonly performed orthopaedic procedures in three major metropolitan teaching hospitals in southwest Sydney, Australia. The hospitals were chosen as they are from a single large health district, but have different roles: one general hospital (mixed minor elective and trauma), one hospital with a large elective orthopaedic caseload, and one with a large trauma orthopaedic workload. Data pertaining to orthopaedic procedures conducted in 2009, 2010 and 2011 were extracted from an electronic clinical database recording details of all procedures undertaken in the operating theatres of these hospitals. All data pertaining to patient records and/or information was de-identified prior to extraction and analysis. Operative procedures - defined as any procedure conducted in an operating theatre by an orthopaedic team that involved penetration of the skin - were identified, grouped under general headings and ranked according to frequency (Appendix S1). Procedure groupings were determined according to anatomical site and/or nature of the procedure performed. For example, “unilateral total arthroplasty of the hip”, “bilateral total arthroplasty of the hip”, “revision of total arthroplasty of the hip” and “revision of partial arthroplasty of the hip” were grouped under the general heading “hip arthoplasty”. In order to obtain a representative sample, the procedures that comprised the top 95% of the total volume of procedures performed (according to frequency) were included in this study.

### Identification and Selection of RCTs

A search of the Cochrane Central Register of Controlled Trials (CENTRAL), the Cochrane Database of Systematic Reviews (CDSR) and the Database of Abstracts of Reviews of Effects (DARE) was performed to identify RCTs examining each operative procedure. CENTRAL, CDSR and DARE were chosen as these databases index the vast majority of published (and many unpublished) RCTs [21]. For each procedure, a search strategy was formulated with the help of a medical librarian, incorporating an RCT filter used by the Scottish Intercollegiate Guidelines Network. Retrieved abstracts were independently examined by two reviewers, and included if the study was a full-text RCT comparing the operative procedure to non-operative treatment(s). We defined an RCT as a study in which participants are randomly (that is, by chance) assigned to one of two or more treatment arms of the clinical trial [22]. RCTs comparing different operative modalities, for instance mobile versus fixed bearing implants for total knee arthroplasty, were excluded as the primary aim of the study was to determine the evidence base for operative interventions compared to non-operative alternatives. RCTs were also excluded after full-text assessment if the method of participant allocation was quasi-randomised (e.g. by date of birth or alternation), if the publication related to a follow-up study utilising a cohort from previously published RCTs or if published after December 2010. Excluding recent RCTs increased the likelihood that the study was available at the time of the intervention.

### Data Extraction and Categorisation

Included RCTs were independently assessed in their entirety by two reviewers, and the sample size and population, the specific interventions being compared, primary outcome, primary outcome findings, secondary outcomes, findings of secondary outcomes and the authors' conclusions were identified. Given that our objective was to determine whether *any* RCT evidence may be found for commonly performed orthopaedic procedures, we crudely categorized each RCT as being either 'supportive’ or 'not supportive’ of operative treatment using the following criteria:

- For RCTs where a primary outcome was stated, and the findings demonstrated a statistically significant result in favour of operative treatment for the stated primary outcome – regardless of the results of any stated secondary outcome(s) – the study was categorised as being 'supportive of operative treatment'.
- For RCTs where a primary outcome was stated, and the findings demonstrated a statistically significant result in favour of the non-operative treatment or where no significant difference was shown for the stated primary outcome, the study was categorised as being 'not supportive of operative treatment'.
- For RCTs where a primary outcome was not stated but several outcomes were measured, and where statistically significant results were demonstrated across all measured outcomes in favour of operative treatment, the study was categorized as “supportive of operative treatment”.
- For RCTs where a primary outcome was not stated but several outcomes were measured, and where the measured outcomes did not consistently demonstrate statistically significant results in favour of operative treatment, the study was categorised as 'not supportive of operative treatment'".

Discrepancies were resolved by discussion and arbitrated by a third author if necessary.

### Risk of Bias Assessment

A risk of bias analysis was conducted using the Cochrane Collaboration's Risk of Bias tool [23]. The tool was applied to extract information for six risk of bias categories: random sequence generation, allocation concealment, blinding of participants and personnel, blinding of outcome assessment, attrition bias and selective reporting. For each category, the risk of bias was categorised into high, unclear, or low. An RCT was assessed to be at low risk of bias based on its performance across two domains – allocation concealment and blinding of outcome assessment. These domains were chosen for bias assessment as the significance of good allocation concealment and outcome assessment blinding in minimising bias and, in particular, overestimation of treatment effect is well supported by empirical evidence [24], [25]. While empirical evidence also exists to support the significance of adequate blinding of participants in reducing exaggeration of estimated treatment effects [25] the inherent difficulty of blinding participants in surgical RCTs necessitated the exclusion of this domain in our assessment. Random sequence generation was not included in the final assessment as the exclusion criteria for quasi-randomisation meant that most of the included RCTs performed well within this domain. In contrast to the first four domains, empirical evidence to support the significance of incomplete outcome data on RCT bias is lacking and mainly driven by theoretical considerations [26], [27], and was therefore not included in our assessment.

### Data Analysis

Results were analysed and presented as:

- The proportion of the total volume of procedures that were assessed by RCTs;
- The proportion of the total volume of procedures that were:
  - *supported* by at least one RCT
  - *supported* by at least one low risk of bias RCT
  - *supported* by at least one RCT with sample size greater than or equal to the median sample size of included RCTs

Primary analyses were conducted using total procedure volume, as this was a more accurate reflection of surgical volume. Secondary analyses were conducted using procedure type, rather than total procedure volume; this analysis would highlight which procedure types were supported by high-level evidence.

## Results

**Figure 1:**
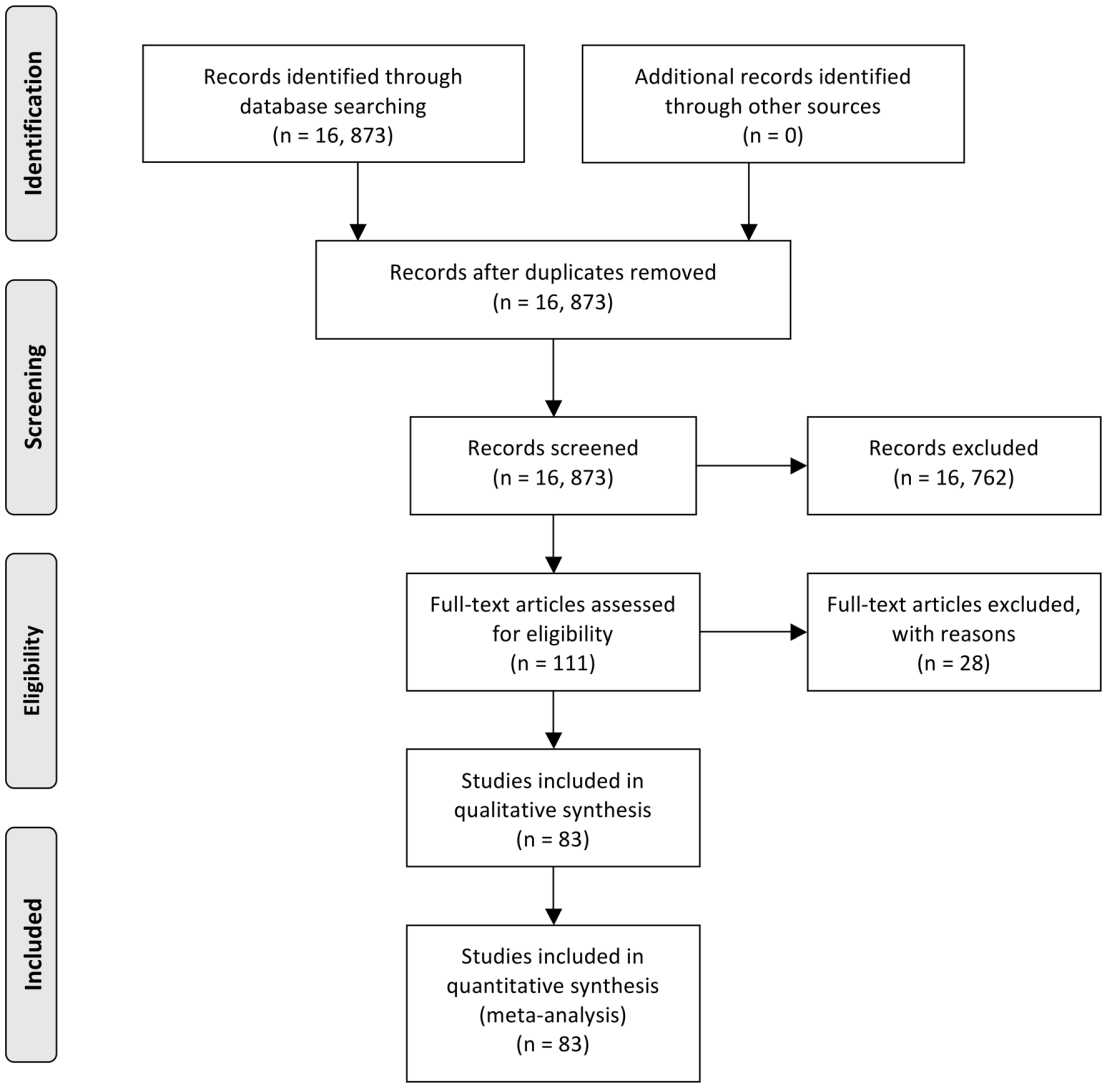
Flow diagram of searches executed, abstracts screened, full-texts screened and RCTs included.

A total volume of 9,392 orthopaedic procedures were performed across the three hospitals in the years 2009, 2010 and 2011 comprising 91 operative procedure categories. 94.6% (8,886 procedures) of the total volume was represented by 32 operative procedure categories. This group was used as the basis for this study. The 10 most commonly performed procedures comprised 80.6% of the 8,886 procedures included in the study (Table 1). 32 different search strategies were executed on CENTRAL, CDSR and DARE databases for dates up to the end of 2010. The search strategies are outlined in Appendix S2. The flowchart of records retrieved from CENTRAL, CDSR and DARE and the screening process to obtain the included articles relevant to each procedure are provided in Table 2. A total of 16,873 abstracts were screened, and of these 83 RCTs were included (Figure 1).

**Table 1:** 10 most commonly performed orthopaedic surgical procedures in 2009, 2010, 2011 ordered according to frequency.

Procedure:		Number of procedures performed
1	Knee arthroscopy	1349
2	Knee arthroplasty	1023
3	Hip arthroplasty	917
4	Removal/debridement/wound cleaning	775
5	Internal fixation of proximal or shaft fracture of the femur	766
6	Internal fixation of distal radius fracture	765
7	Removal of implants	697
8	Ankle fracture fixation	435
9	Acromioplasty repair of rotator cuff	237
10	Shoulder arthroscopy	202
Total		7166

**Table 2:** Flow of RCT screening, inclusion and surgical procedure support for each search executed on CENTRAL/CDSR/DARE on 32 unique procedures.

Procedure:		Number of procedures performed	Number of articles retrieved from CENTRAL/CDSR/DARE	Number of RCTs included after title/abstract screening	Number of RCTs included after full text screening	Number of RCTs supporting surgical procedure	Number of RCTs not supporting surgical procedure	Number of low risk of bias RCTs supporting surgical procedure	Number of RCTs with sample size ≥ 72 supporting surgical procedure
1	Knee arthroscopy	1349	861	12	10	1	9	1	1
2	Knee arthroplasty	1023	1798	0	0	-	-	-	-
3	Hip arthroplasty	917	771	0	0	-	-	-	-
4	Removal/debridement/wound cleaning	775	1967	0	0	-	-	-	-
5	Internal fixation of proximal or shaft fracture of the femur	766	862	2	2	0	2	0	1
6	Internal fixation of distal radius fracture	765	683	14	11	2	9	0	1
7	Removal of implants	697	859	0	0	-	-	-	-
8	Ankle fracture fixation	435	1028	9	7	2	5	0	1
9	Acromioplasty repair of rotator cuff	237	171	8	5	1	4	1	1
10	Shoulder arthroscopy	202	224	0	0	-	-	-	-
11	Open reduction of fracture of shaft of tibia with internal fixation	170	337	4	4	0	4	0	2
12	Osteotomy	169	560	2	2	1	1	0	0
13	Open reduction of joint dislocation (shoulder, acromioclavicular & patella respectively)	157	120, 14, 22	9, 3, 6	6, 3, 4	5, 0, 1	1, 3, 3	2, 0, 0	1, 0, 0
14	Knee, repair of cruciate ligament	157	662	6	3	0	3	0	1
15	Tibia, plateau of, medial or lateral fracture, open	100	341	0	0	-	-	-	-
16	Repair of achilles tendon rupture	89	281	13	9	0	9	0	2
17	Humerus, distal, treatment of fracture by open reduction	87	191	3	1	0	1	0	0
18	Olecranon, treatment of fracture by open reduction (intern fix)	82	433	0	0	-	-	-	-
19	Arthroscopy of ankle	77	48	0	0	-	-	-	-
20	Joint arthrodesis	76	742	1	1	0	1	0	0
21	Abscess drainage	69	988	5	5	1	4	0	0
22	Clavicle, treatment of fracture, open reduction	61	89	4	3	1	2	0	1
23	Patella, treatment fracture, by internal fixation open reduction	53	142	0	0	-	-	-	-
24	Humerus, proximal, treatment of fracture, open reduction	53	191	3	2	0	2	0	0
25	Amputation	48	678	0	0	-	-	-	-
26	Foot (not talus or calcaneus) fracture fixation	47	285	0	0	-	-	-	-
27	Acetabulum, treatment of fracture by open reduction	42	443	0	0	-	-	-	-
28	Excision of ganglion	40	198	0	0	-	-	-	-
29	Wedge resection of ingrown toenail	39	301	0	0	-	-	-	-
30	Release of carpal tunnel	37	397	6	5	4	1	0	3
31	Shoulder Arthroplasty	37	99	0	0	-	-	-	-
32	Fasciotomy	30	87	0	0	-	-	-	-
Total		8886	16873	110	83	19	64	3	15

Of the 83 RCTs included, 22.9% (19/83) were classified as supportive of operative intervention. The median sample size of the included RCTs was 72. A list of included and excluded studies is found in Appendix S3; characteristics of included studies are summarised in Appendix S4.

### Proportion of Total Procedure Volume with RCT Evidence

**Figure 2:**
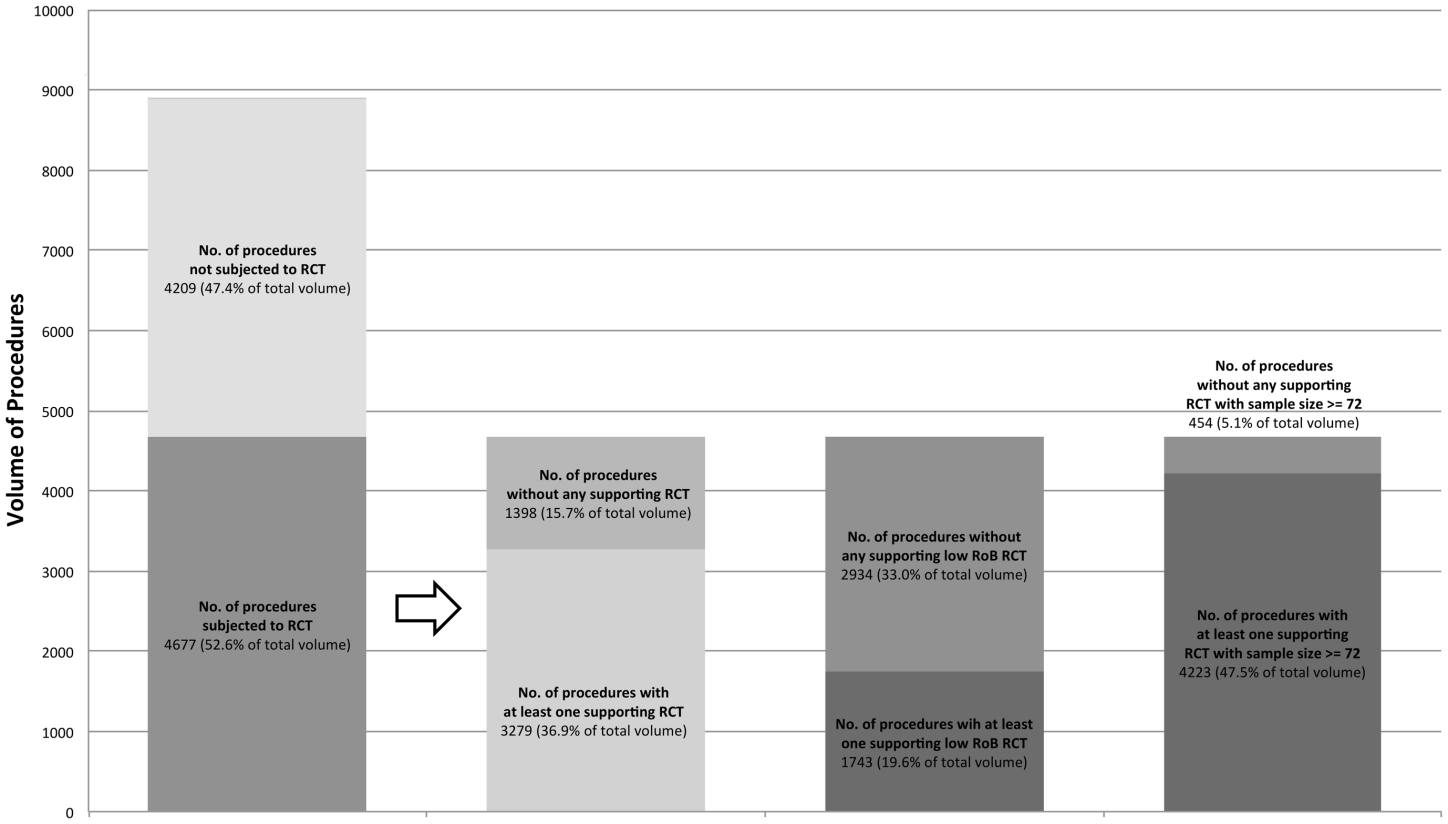
Procedure Volume versus Degree of RCT Evidence and Support.

52.6% (4,677/8886) of the total volume of procedures were subjected to an RCT comparing operative to non-operative treatment. 36.9% (3279/8886) of total volume of procedures had at least one RCT supporting operative intervention. When analysed according to risk of bias assessments, 19.6% (1743/8886) of total volume of procedures had at least one low risk of bias RCT supporting operative intervention. When analysed against sample size, 47.5% (4223/8886) of total volume of procedures had at least one RCT with sample size greater than or equal to 72 supporting operative intervention (Figure 2).

### Proportion of Procedure Types with RCT Evidence

**Figure 3:**
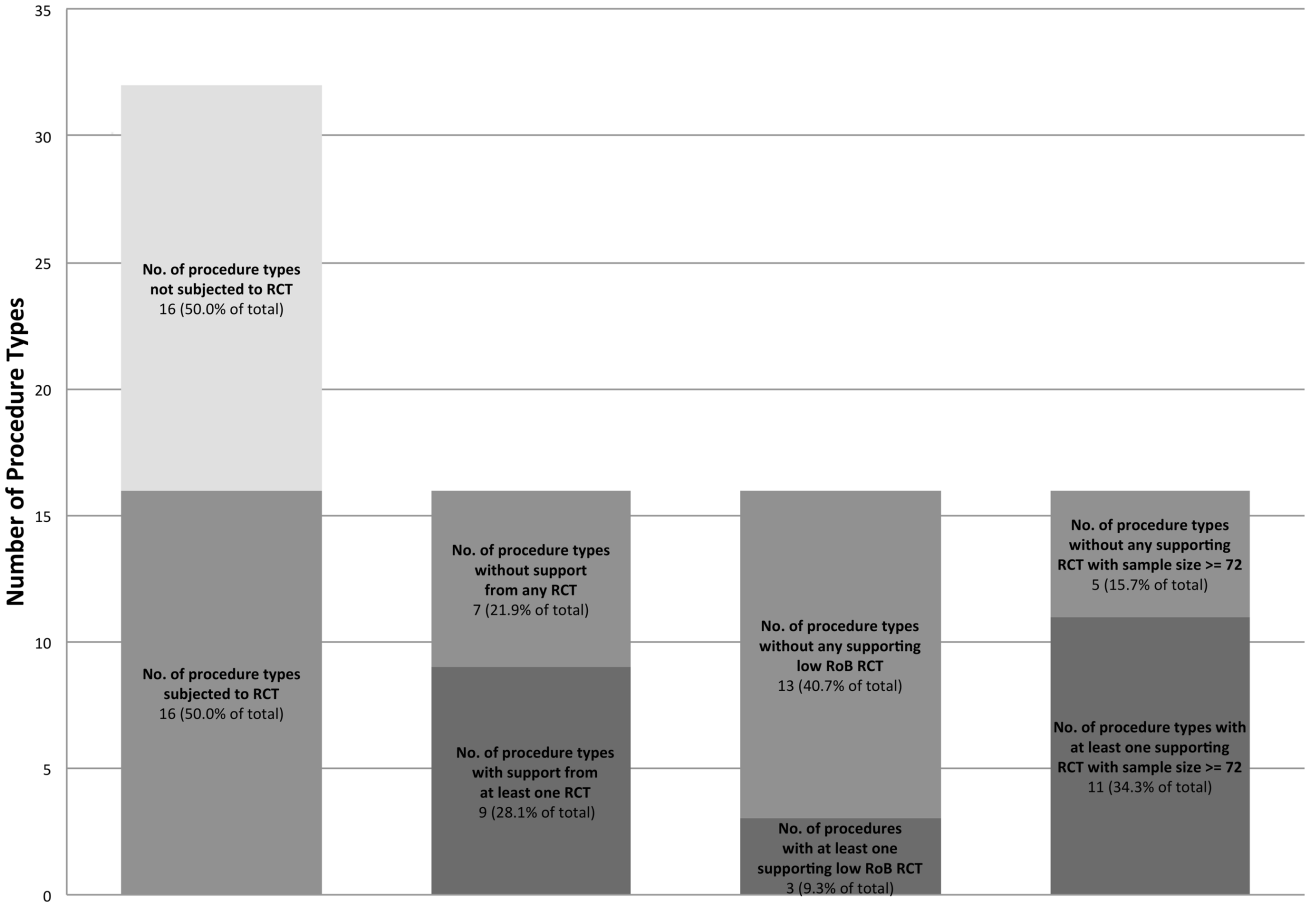
Procedure Type versus Degree of RCT Evidence and Support.

Of the 32 procedure types comprising 94.6% of the total volume of procedures, 16 (50%) had RCTs comparing the procedure to non-operative treatment. 28.1% (13/32) procedure types had at least one RCT supporting operative intervention. When analysed according to risk of bias, 9.3% (3/32) procedure types had at least one low risk of bias RCT supporting the operative intervention. 34.3% (11/32) procedure types had at least one RCT with sample size greater or equal to 72 supporting operative intervention (Figure 3).

### Risk of Bias Summary

12 of the 83 included RCTs were assessed as having low risk of bias across the two domains: allocation concealment and blinding of outcome assessment. 26 RCTs were assessed as having unclear risk of bias and 45 as having high risk of bias across the two domains. Four of the 12 RCTs assessed as having low risk of bias (Arden 2008, Moosmayer 2010, Wintzell 1999 and Wintzell 2000) were supportive of operative treatment.

**Figure 4:**
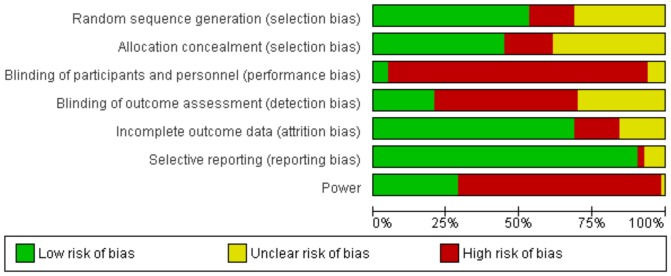
Risk of bias assessment summary for 83 included RCTs using the Cochrane Collaboration's risk of bias tool.

A risk of bias summary for each domain is depicted in Figure 4. Overall, studies performed well in dealing with biases related to selective reporting and attrition, but only half of all trials had adequate methods related to randomisation. There was generally a high risk of bias in the domains of blinding, which reflects the difficulty of blinding trials involving operative interventions, particularly when comparing to non-operative treatments. Risk of bias data for each of the 83 included RCTs is included in Appendix S5.

## Discussion

We reviewed the available RCT evidence in relation to commonly performed orthopaedic procedures in the years 2009, 2010 and 2011 to establish what proportion of these procedures was supported by RCT evidence. We found that 52.6% of the total volume of orthopaedic procedures performed had been compared to non-operative treatments in at least one RCT, and that 36.9% of the total volume of procedures performed was supported by at least one RCT in favour of the operative treatment. Where RCT evidence was available, the quality of that evidence was largely deemed sub-optimal due to inherent biases in the trial methodology.

While the proportion of RCTs in surgical publications has increased over recent decades, our findings suggest that the proportion of surgical interventions supported by quality RCT-level evidence remains low. In general, this is in keeping with the findings of earlier studies.

Howes et al conducted a prospective study looking at the evidence-base for surgical decisions made on 100 patients admitted to a general surgical ward in a tertiary teaching hospital. The literature concerning the efficacy of each treatment was reviewed and it was found that while 95% of the surgical interventions studied were evidence-based, only 24% were based on RCT-level evidence, defined as at least one RCT in favour of the surgical approach [6]. Similar results were reported in a study by Baraldini et al who analysed the levels of evidence for surgical procedures performed on 49 patients admitted over a 4-week period to a paediatric surgical unit. The study found that while 97% of the procedures performed were evidence based, only 26% were based on RCT-level evidence where there was at least one study in favour of surgery [8]. Findings from a prospective review by Kenny et al of 281 paediatric patients who all received primary surgical interventions again showed a low proportion (11%) of RCT-level evidence for the interventions [17]. These figures are substantially lower than the values of 53% and 57% obtained in general medicine, as reported by Ellis et al and Michaud et al, respectively [5], [28].

For surgical procedures performed commonly, it is not unreasonable to expect decision-making to be based on a high level of scientific evidence. Our findings, however, show that despite 52.6% of the total procedure volume being subjected to at least one RCT, only 19.6% of performed procedures had at least one low risk of bias RCT supporting the operative treatment over non-operative alternatives. Similarly, only a minority of the procedure types were supported by higher quality RCT evidence in favour of the surgical procedure. These comparatively low figures are in part explained by our methodology. In contrast to the three studies highlighted above, our study analysed the results according to the quality of RCT evidence. If the figures obtained in the current study were interpreted using the same definition as that of the previous studies, this would result in 36.9% of total volume of procedures having support from at least one RCT. While this figure may seem comparatively favourable, we consider our assessment to be a more accurate reflection of available evidence.

Despite the methodological differences between studies, the common finding is that there is a paucity of high-level evidence to support a large volume of surgical procedures. This disconnect between evidence and practice may be explained by several factors, including clinician bias. Katz, in his ethnography on the culture of surgeons, contended that “surgeons have been resistant to accepting new scientific findings and applying them to their practice" [29]. The lack of generalisability of randomised controlled trial findings to individual patients is often proposed as a reason for the surgeon's reticence to accept RCT evidence [30]. However well-conducted the trial, the rigidity of RCT design means that the findings can only suggest what was more effective for a specific group of patients with a particular condition, but not whether this evidence is applicable to a particular case or to an indiv*i*dual patient [30]. An RCT is also considered by surgeons to be too simplistic to adequately assess the complex nature of surgical interventions [32]. The various components of a surgical intervention in the pre-operative, intra-operative and post-operative stages all significantly influence the final outcome - the complexity of which cannot be adequately accounted for by an RCT [31]. That the quality of the RCT evidence that is available is generally poor may also serve to explain the lack of acceptance.

The 83 included RCTs had variable levels of bias. The risk of bias summary for each surgical procedure consistently showed poor scores in the performance and outcome detection categories of bias due to difficulties with blinding the surgical intervention. Furthermore, a quarter of studies reported poorly on their randomisation strategy while only a handful performed sample size calculations for power. The only category that was consistently adequate across the included RCTs was follow-up. Of the two included domains, RCTs performed most poorly in blinding of outcome assessment. Just 17 studies specifically attempted to address this potential source of detection bias in their methodology and was the largest avoidable contributor to the overall high risk of bias across the included studies.

When RCTs were analysed against sample size, less than half the total volume of procedures were supported by RCTs with a sample size greater than the median of 72. Though inadequate sample size does not directly influence study bias, underpowered studies are more likely to have a greater rates of type II errors in which studies fail to detect statistically significant treatment effects. An investigation by Lochner et al into the rates of type II errors of randomised trials involving orthopaedic fracture care showed higher than accepted levels of type II errors and, low mean level of study power [33]. The high proportion of underpowered orthopaedic RCTs may be one of the reasons for the low support rate seen in our study.

We acknowledge the limitations of our study. Not subdividing the RCT evidence by indication for the specific procedures in our analysis (for example, the removal of implants for pain as opposed to other indications, or cruciate ligament reconstruction for an isolated tear as opposed to a multi-ligamentous injury) is a potential limitation of this study as the RCT evidence support for a procedure may be restricted to specific indications. Given the primary aim of the study was to assess potential differences between available RCT evidence and current clinical practice, an analysis subdividing each of the included procedure groups for indication would have contributed little value to the primary outcome. As our criteria allowed inclusion of supportive RCTs for any indication, however, it is likely that there was a potential overestimation of RCT support for any procedure group. Our study investigated RCT-level evidence exclusively as this study design provides the least biased evidence for the efficacy and safety of an intervention though its ability to deal with confounding [4]. This does not imply that every operative procedure requires RCT-level evidence to support its efficacy over a non-operative alternative. Large, well-designed prospective cohort studies can adequately minimise bias comparable to that of RCTs [28] and therefore have acceptable levels of validity in cases where an RCT may not be feasible [34], [35].

While improvements have occurred in the quantity and to a lesser extent, the quality of surgical research over the past several decades, this study confirms that, consistent with other surgical specialities, the majority of orthopaedic surgical interventions are not based on RCT evidence. The findings of this study are reflections of the current disconnect between trial evidence and orthopaedic surgical practice and its comparison to other specialities. These findings support the need for better quality RCTs to evaluate the indications for orthopaedic procedures and stakeholder discussions about the lack of support for many procedures currently being performed.

## Supporting Information

Appendix S1 **List of procedures performed ordered according to frequency.**
(DOCX)

Appendix S2 **Syntax of search strategies for each operative procedure category.**
(DOCX)

Appendix S3 **List of included and excluded RCTs after full-text assessment.**
(DOCX)

Appendix S4 **Characteristics of included RCTs.**
(DOCX)

Appendix S5 **Risk of Bias assessment of 83 included RCTs using the Cochrane Collaboration's Risk of Bias tool.**
(PDF)

Checklist S1 (DOC)
