# Correction: Randomised Trial Support for Orthopaedic Surgical Procedures

**DOI:** 10.1371/journal.pone.0144682

**Published:** 2015-12-11

**Authors:** Hyeung C. Lim, Sam Adie, Justine M. Naylor, Ian A. Harris

The image for Fig 2 is incorrect. Please see the corrected Fig 2 here.

**Fig 2:**
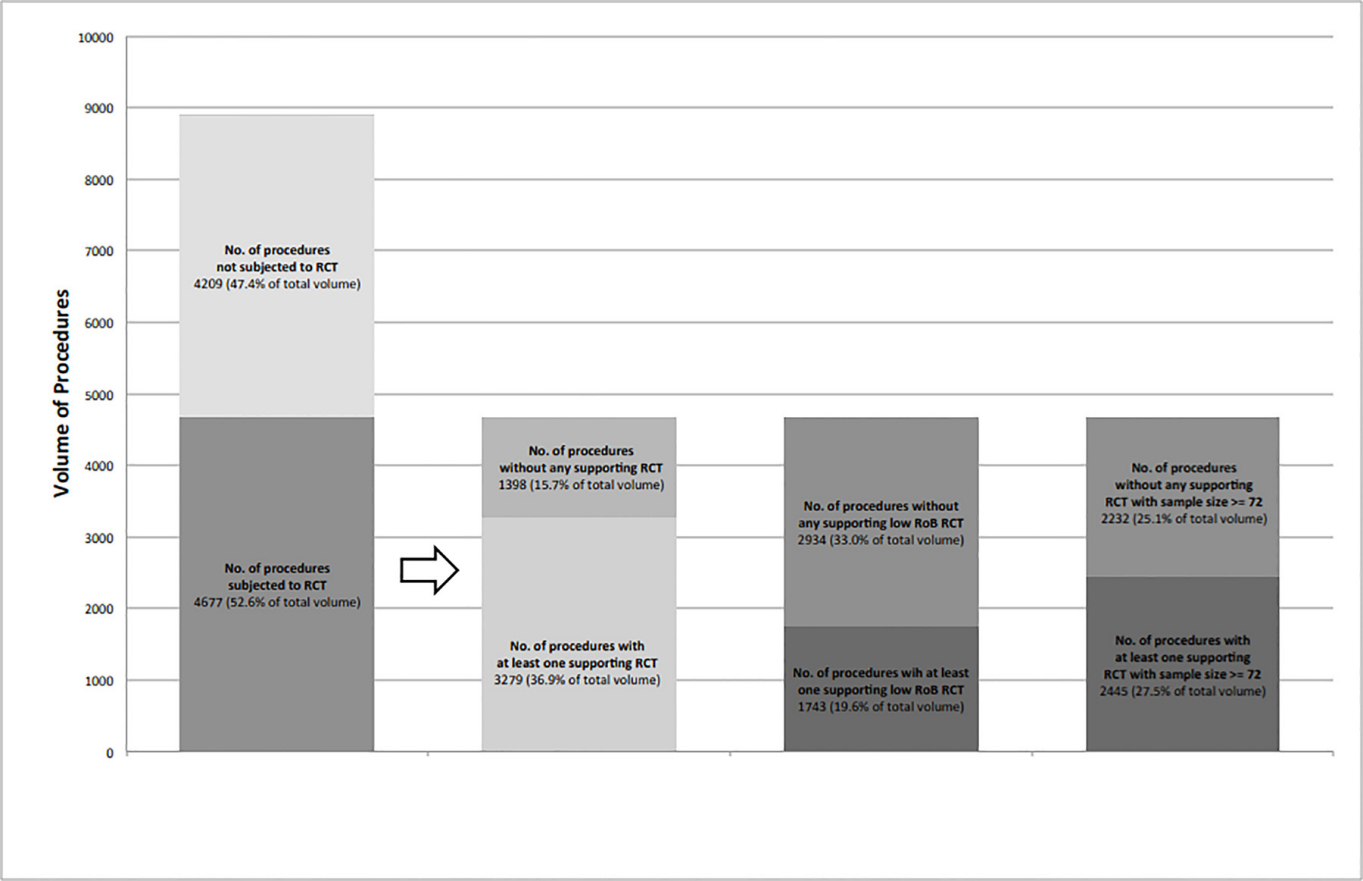
Procedure Volume versus Degree of RCT Evidence and Support.

The image for Fig 3 is incorrect. Please see the corrected Fig 3 here.

**Fig 3:**
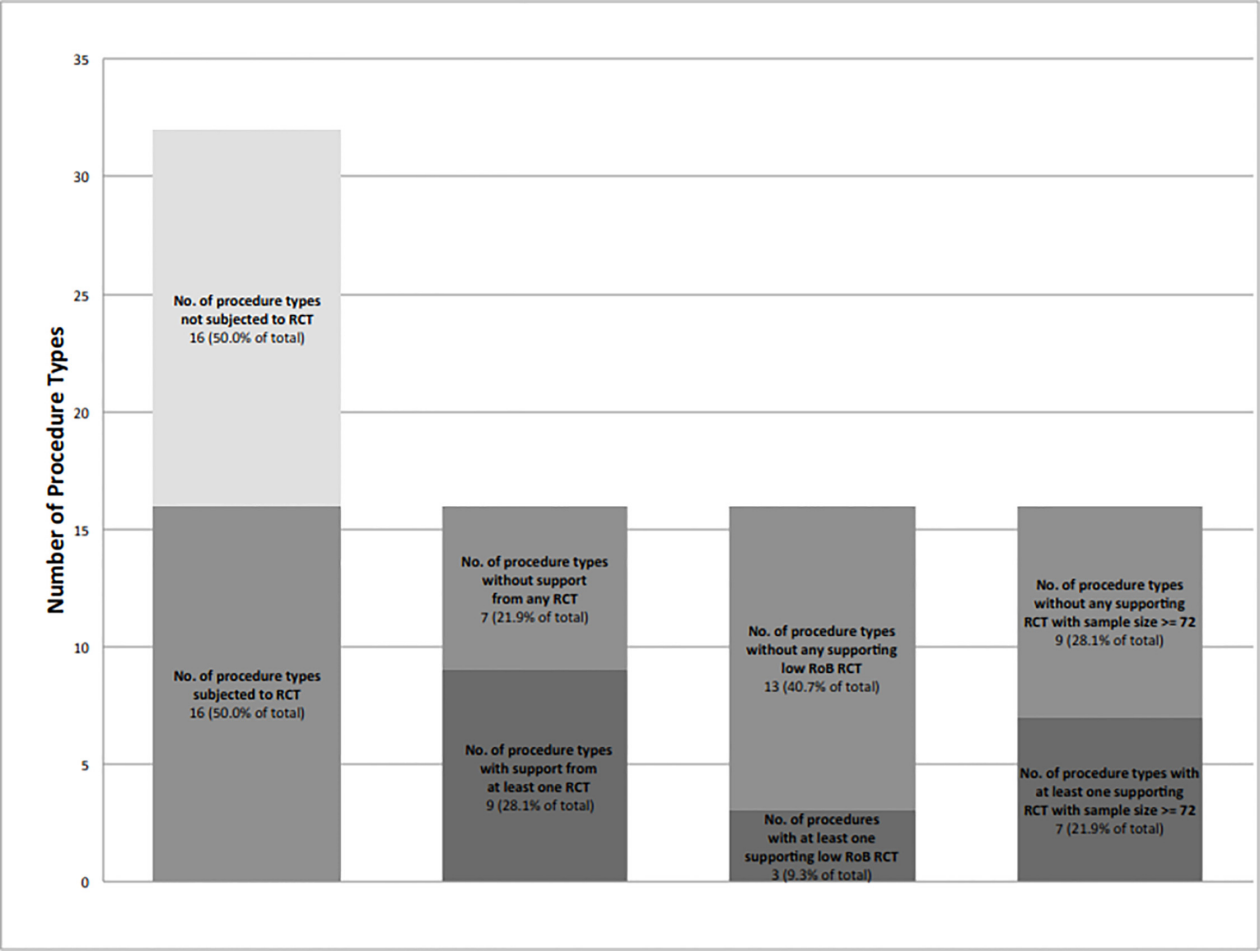
Procedure Type versus Degree of RCT Evidence and Support.

There are errors in the “Number of RCTs with sample size ≥ 72 supporting surgical procedure” column of Table 2. Please see the corrected Table 2 here.

**Table 2:** Flow of RCT screening, inclusion and surgical procedure support for each search executed on CENTRAL/CDSR/DARE on 32 unique procedures.

Procedure:		Number of procedures performed	Number of articles retrieved from CENTRAL/ CDSR/ DARE	Number of RCTs included after title/ abstract screening	Number of RCTs included after full text screening	Number of RCTs supporting surgical procedure	Number of RCTs not supporting surgical procedure	Number of low risk of bias RCTs supporting surgical procedure	Number of RCTs with sample size ≥ 72 supporting surgical procedure
1	Knee arthroscopy	1349	861	12	10	1	9	1	1
2	Knee arthroplasty	1023	1798	0	0	-	-	-	-
3	Hip arthroplasty	917	771	0	0	-	-	-	-
4	Removal/ debridement/ wound cleaning	775	1967	0	0	-	-	-	-
5	Internal fixation of proximal or shaft fracture of the femur	766	862	2	2	0	2	0	0
6	Internal fixation of distal radius fracture	765	683	14	11	2	9	0	0
7	Removal of implants	697	859	0	0	-	-	-	-
8	Ankle fracture fixation	435	1028	9	7	2	5	0	1
9	Acromioplasty repair of rotator cuff	237	171	8	5	1	4	1	1
10	Shoulder arthroscopy	202	224	0	0	-	-	-	-
11	Open reduction of fracture of shaft of tibia with internal fixation	170	337	4	4	0	4	0	0
12	Osteotomy	169	560	2	2	1	1	0	1
13	Open reduction of joint dislocation (shoulder, acromioclavicular & patella respectively)	157	120, 14, 22	9, 3, 6	6, 3, 4	5, 0, 1	1, 3, 3	2, 0, 0	1, 0, 0
14	Knee, repair of cruciate ligament	157	662	6	3	0	3	0	0
15	Tibia, plateau of, medial or lateral fracture, open	100	341	0	0	-	-	-	-
16	Repair of achilles tendon rupture	89	281	13	9	0	9	0	0
17	Humerus, distal, treatment of fracture by open reduction	87	191	3	1	0	1	0	0
18	Olecranon, treatment of fracture by open reduction (intern fix)	82	433	0	0	-	-	-	-
19	Arthroscopy of ankle	77	48	0	0	-	-	-	-
20	Joint arthrodesis	76	742	1	1	0	1	0	0
21	Abscess drainage	69	988	5	5	1	4	0	0
22	Clavicle, treatment of fracture, open reduction	61	89	4	3	1	2	0	1
23	Patella, treatment fracture, by internal fixation open reduction	53	142	0	0	-	-	-	-
24	Humerus, proximal, treatment of fracture, open reduction	53	191	3	2	0	2	0	0
25	Amputation	48	678	0	0	-	-	-	-
26	Foot (not talus or calcaneus) fracture fixation	47	285	0	0	-	-	-	-
27	Acetabulum, treatment of fracture by open reduction	42	443	0	0	-	-	-	-
28	Excision of ganglion	40	198	0	0	-	-	-	-
29	Wedge resection of ingrown toenail	39	301	0	0	-	-	-	-
30	Release of carpal tunnel	37	397	6	5	4	1	0	3
31	Shoulder Arthroplasty	37	99	0	0	-	-	-	-
32	Fasciotomy	30	87	0	0	-	-	-	-
**Total**		**8886**	**16873**	**110**	**83**	**19**	**64**	**3**	**9**
