# Supplementary material for: Randomised Trial Support for Orthopaedic Surgical Procedures
Source: PLoS One. 2014 Jun 13;9(6):e96745. doi: 10.1371/journal.pone.0096745 (PMC4057075; doi:10.1371/journal.pone.0096745)
Supplement: Appendix S1 — List of procedures performed ordered according to frequency. (DOCX) [file pone.0096745.s001.docx]

**APPENDIX S1**

**List of procedures performed ordered according to frequency**

| **Procedure:** | | | **Hospital 1** | | | | | **Hospital 2** | | | | | | | **Hospital 3** | | | | | | | **Total** | |
| --- | --- | --- | --- | --- | --- | --- | --- | --- | --- | --- | --- | --- | --- | --- | --- | --- | --- | --- | --- | --- | --- | --- | --- |
|  |  |  | **2009** | **2010** | | **2011** | | **2009** | **2010** | | | **2011** | | | **2009** | | | **2010** | | **2011** | |  |  |
| **1** | | Knee arthroscopy | 222 | 184 | | 153 | | 211 | 212 | | | 237 | | | 38 | | | 51 | | 41 | | 1349 | |
| **2** | | Knee arthroplasty | 0 | 0 | | 0 | | 294 | 401 | | | 316 | | | 4 | | | 4 | | 4 | | 1023 | |
| **3** | | Hip arthroplasty | 25 | 31 | | 37 | | 150 | 198 | | | 188 | | | 108 | | | 85 | | 95 | | 917 | |
| **4** | | Removal/debridement/wound cleaning | 16 | 55 | | 79 | | 0 | 0 | | | 0 | | | 205 | | | 171 | | 249 | | 775 | |
| **5** | | Internal fixation of proximal or shaft fracture of the femur | 74 | 45 | | 67 | | 0 | 0 | | | 0 | | | 179 | | | 194 | | 207 | | 766 | |
| **6** | | Internal fixation of distal radius fracture | 105 | 151 | | 135 | | 1 | 0 | | | 0 | | | 129 | | | 128 | | 116 | | 765 | |
| **7** | | Removal of implants | 82 | 74 | | 91 | | 22 | 10 | | | 6 | | | 154 | | | 124 | | 134 | | 697 | |
| **8** | | Ankle fracture fixation | 59 | 51 | | 66 | | 1 | 0 | | | 1 | | | 76 | | | 97 | | 84 | | 435 | |
| **9** | | Acrominoplasty repair of rotator cuff | 31 | 28 | | 11 | | 3 | 72 | | | 78 | | | 4 | | | 5 | | 5 | | 237 | |
| **10** | | Shoulder arthroscopy | 4 | 16 | | 17 | | 72 | 47 | | | 35 | | | 3 | | | 3 | | 5 | | 202 | |
| **11** | | Open reduction of fracture of shaft of tibia with internal fixation | 7 | 10 | | 17 | | 0 | 0 | | | 0 | | | 55 | | | 40 | | 41 | | 170 | |
| **12** | | Osteotomy | 13 | 11 | | 25 | | 20 | 23 | | | 28 | | | 12 | | | 16 | | 21 | | 169 | |
| **13** | | Open reduction of joint dislocation | 20 | 19 | | 13 | | 5 | 9 | | | 9 | | | 23 | | | 23 | | 36 | | 157 | |
| **14** | | Knee, repair of cruciate ligament | 13 | 14 | | 24 | | 20 | 27 | | | 37 | | | 8 | | | 6 | | 8 | | 157 | |
| **15** | | Tibial plateau (medial or lateral fracture), open reduction | 13 | 8 | | 4 | | 0 | 0 | | | 0 | | | 25 | | | 33 | | 17 | | 100 | |
| **16** | | Repair of achilles tendon rupture | 15 | 17 | | 11 | | 0 | 0 | | | 0 | | | 21 | | | 17 | | 8 | | 89 | |
| **17** | | Humerus, distal, treatment of fracture by open reduction | 5 | 4 | | 6 | | 0 | | | 0 | | | 0 | | | 15 | | 36 | | 21 | | 87 |
| **18** | | Olecranon, treatment of fracture by open reduction (intern fix) | 3 | 6 | | 18 | | 0 | | | 0 | | | 0 | | | 16 | | 25 | | 14 | | 82 |
| **19** | | Arthroscopy of ankle | 9 | 9 | | 20 | | 3 | | | 2 | | | 7 | | | 4 | | 9 | | 14 | | 77 |
| **20** | | Joint arthrodesis | 5 | 7 | | 18 | | 7 | | | 8 | | | 10 | | | 8 | | 6 | | 7 | | 76 |
| **21** | | Abscess drainage | 3 | 18 | | 13 | | 0 | | | 1 | | | 0 | | | 11 | | 11 | | 12 | | 69 |
| **22** | | Clavicle, treatment of fracture, open reduction wit | 7 | | 5 | | 14 | 0 | | | 0 | | | 0 | | | 11 | | 14 | | 10 | | 61 |
| **23** | | Patella, treatment fracture, by internal fixation open reduction | 6 | | 5 | | 4 | 0 | | | 0 | | | 0 | | | 18 | | 11 | | 9 | | 53 |
| **24** | | Humerus, proximal, treatment of fracture , open reduction | 6 | | 5 | | 14 | 0 | | | 0 | | | 0 | | | 11 | | 12 | | 5 | | 53 |
| **25** | | Amputation | 1 | | 2 | | 6 | 0 | | | 2 | | | 5 | | | 5 | | 10 | | 17 | | 48 |
| **26** | | Foot (not talus or calcaneus) fracture fixation | 2 | | 9 | | 5 | 0 | | | 0 | | | 0 | | | 10 | | 13 | | 8 | | 47 |
| **27** | | Acetabulum, treatment of fracture by open reduction | 0 | | 1 | | 2 | 0 | | | 0 | | | 0 | | | 16 | | 11 | | 12 | | 42 |
| **28** | | Excision of ganglion | 5 | | 3 | | 8 | 0 | | | 5 | | | 10 | | | 2 | | 2 | | 5 | | 40 |
| **29** | | Wedge resection of ingrown toenail | 11 | | 5 | | 2 | 11 | | | 1 | | | 2 | | | 2 | | 4 | | 1 | | 39 |
| **30** | | Release of carpal tunnel | 5 | | 5 | | 6 | 0 | | | 10 | | | 9 | | | 0 | | 0 | | 2 | | 37 |
| **31** | | Shoulder arthroplasty | 2 | | 2 | | 3 | 6 | | | 10 | | | 3 | | | 4 | | 2 | | 5 | | 37 |
| **32** | | Fasciotomy | 1 | | 0 | | 4 | 0 | | | 0 | | | 0 | | | 13 | | 7 | | 5 | | 30 |
| **33** | | Bone grafting with internal fixation | 1 | | 3 | | 1 | 2 | | | 1 | | | 1 | | | 7 | | 8 | | 5 | | 29 |
| **34** | | Ulna, distal end, treatment of fracture by open re | 1 | | 3 | | 3 | 0 | | 0 | | | 0 | | | 3 | | | 13 | | 6 | | 29 |
| **35** | | Radius, treatment of fracture of head or neck, open | 2 | | 4 | | 4 | 0 | | 0 | | | 0 | | | 5 | | | 5 | | 8 | | 28 |
| **36** | | Lavage of hip, knee, ankle | 0 | | 0 | | 0 | 0 | | 1 | | | 1 | | | 7 | | | 10 | | 9 | | 28 |
| **37** | | Stabilisation of shoulder for recurrent dislocation | 1 | | 1 | | 4 | 3 | | 2 | | | 5 | | | 2 | | | 2 | | 4 | | 24 |
| **38** | | Release of tendon sheath of hand (trigger finger) | 1 | | 3 | | 3 | 0 | | 5 | | | 8 | | | 0 | | | 0 | | 3 | | 23 |
| **39** | | Pelvic ring, treatment of fracture by open reduction | 0 | | 0 | | 0 | 0 | | 0 | | | 0 | | | 6 | | | 9 | | 7 | | 22 |
| **40** | | Open reduction intern fix of finger/hand fracture | 3 | | 1 | | 1 | 0 | | 0 | | | 0 | | | 5 | | | 8 | | 3 | | 21 |
| **41** | | Exostosis of small/large bone of foot | 2 | | 2 | | 2 | 2 | | 4 | | | 1 | | | 2 | | | 2 | | 2 | | 19 |
| **42** | | Repair of fingernail or nail bed | 1 | | 1 | | 2 | 0 | | 0 | | | 0 | | | 6 | | | 5 | | 3 | | 18 |
| **43** | | Primary repair of flexor or extensor tendon of foot | 1 | | 2 | | 0 | 0 | | 0 | | | 0 | | | 2 | | | 7 | | 6 | | 18 |
| **44** | | Femur, condylar region treatment of intraarticular fracture | 0 | | 0 | | 2 | 0 | | 0 | | | 0 | | | 0 | | | 12 | | 3 | | 17 |
| **45** | | Open reduction of talus fracture with internal fixation | 1 | | 0 | | 1 | 0 | | 0 | | | 0 | | | 1 | | | 7 | | 6 | | 16 |
| **46** | | Humerus shaft, open reduction | 0 | | 0 | | 0 | 0 | | 0 | | | 0 | | | 6 | | | 4 | | 4 | | 14 |
| **47** | | Arthroscopy of hip | 3 | | 2 | | 3 | 0 | | 1 | | | 3 | | | 1 | | | 0 | | 0 | | 13 |
| **48** | | Lengthening of achilles tendon | 1 | | 0 | | 0 | 0 | | 0 | | | 0 | | | 3 | | | 5 | | 4 | | 13 |
| **49** | | Biopsy of bone | 0 | | 1 | | 0 | 3 | | 0 | | | 0 | | | 3 | | | 3 | | 2 | | 12 |
| **50** | | Skin grafting | 0 | | 0 | | 0 | 0 | | 0 | | | 0 | | | 2 | | | 4 | | 5 | | 11 |
| **51** | | Arthroscopy of elbow | 4 | | 3 | | 2 | 0 | | 1 | | | 0 | | | 0 | | | 0 | | 0 | | 10 |
| **52** | | Injection into joint or other synovial cavity | 0 | | 2 | | 3 | 1 | | 1 | | | 2 | | | 0 | | | 0 | | 0 | | 9 |
| **53** | | Subcutaneous fasciotomy for dupuytren's contracture | 1 | | 1 | | 0 | 3 | | 1 | | | 0 | | | 0 | | | 1 | | 1 | | 8 |
| **54** | | Arthrotomy of elbow | 0 | | 0 | | 1 | 2 | | 3 | | | 1 | | | 0 | | | 0 | | 0 | | 7 |
| **55** | | Elbow arthroplasty | 0 | | 0 | | 0 | 0 | | 0 | | | 0 | | | 2 | | | 1 | | 4 | | 7 |
| **56** | | Arthrotomy of knee | 0 | | 1 | | 1 | 2 | | 2 | | | 1 | | | 0 | | | 0 | | 0 | | 7 |
| **57** | | Aspiration of a joint or other synovial cavity | 1 | | 0 | | 0 | 0 | | 0 | | | 1 | | | 1 | | | 2 | | 2 | | 7 |
| **58** | | Arthrotomy of ankle | 1 | | 1 | | 0 | 1 | | 0 | | | 0 | | | 2 | | | 0 | | 0 | | 5 |
| **59** | | Arthroplasty of ankle | 0 | | 0 | | 0 | 3 | | 2 | | | 0 | | | 0 | | | 0 | | 0 | | 5 |
| **60** | | Excision of benign lesion of skin and subcutaneous | 0 | | 0 | | 1 | 0 | | 0 | | | 0 | | | 2 | | | 0 | | 2 | | 5 |
| **61** | | Application of external fixation device, not elsewhere specified | 1 | | 0 | | 0 | 0 | | 0 | | | 0 | | | 0 | | | 2 | | 2 | | 5 |
| **62** | | Fibula, treatment of fracture | 0 | | 2 | | 0 | 0 | | 0 | | | 0 | | | 0 | | | 1 | | 2 | | 5 |
| **63** | | Transfer of tendon | 0 | | 2 | | 0 | 0 | | 0 | | | 0 | | | 0 | | | 1 | | 2 | | 5 |
| **64** | | Adjustment of ring fixator or similar device | 0 | | 0 | | 0 | 0 | | 0 | | | 0 | | | 3 | | | 1 | | 0 | | 4 |
| **65** | | Total replacement arthroplasty of patellofemoral joint | 0 | | 0 | | 0 | 0 | | 2 | | | 1 | | | 0 | | | 1 | | 0 | | 4 |
| **66** | | Patello-femoral stabilisation | 0 | | 0 | | 1 | 0 | | 3 | | | 0 | | | 0 | | | 0 | | 0 | | 4 |
| **67** | | Forage of neck and/or head of femur | 0 | | 0 | | 1 | 0 | | 1 | | | 1 | | | 0 | | | 1 | | 0 | | 4 |
| **68** | | Arthrotomy of hip | 0 | | 1 | | 0 | 0 | | 0 | | | 0 | | | 0 | | | 2 | | 1 | | 4 |
| **69** | | Patellectomy | 0 | | 0 | | 0 | 2 | | 1 | | | 0 | | | 0 | | | 0 | | 0 | | 3 |
| **70** | | Knee, repair of collateral ligament | 1 | | 0 | | 0 | 0 | | 0 | | | 0 | | | 1 | | | 1 | | 0 | | 3 |
| **71** | | Plantar fasciectomy | 1 | | 1 | | 0 | 1 | | 0 | | | 0 | | | 0 | | | 0 | | 0 | | 3 |
| **72** | | Wrist arthroscopy | 0 | | 0 | | 1 | 0 | | 0 | | | 0 | | | 1 | | | 1 | | 0 | | 3 |
| **73** | | Neurectomy of foot | 1 | | 0 | | 0 | 0 | | 0 | | | 0 | | | 0 | | | 0 | | 2 | | 3 |
| **74** | | Epicondylitis, open operation | 0 | | 0 | | 0 | 0 | | 1 | | | 2 | | | 0 | | | 0 | | 0 | | 3 |
| **75** | | Quadricepsplasty of knee | 0 | | 0 | | 1 | 0 | | 1 | | | 0 | | | 0 | | | 1 | | 0 | | 3 |
| **76** | | Open tenotomy | 0 | | 0 | | 0 | 0 | | 0 | | | 0 | | | 0 | | | 1 | | 2 | | 3 |
| **77** | | Open neurolysis of peripheral nerve | 0 | | 0 | | 0 | 0 | | 0 | | | 0 | | | 1 | | | 1 | | 0 | | 2 |
| **78** | | Stabilisation of elbow | 0 | | 0 | | 0 | 0 | | 0 | | | 0 | | | 0 | | | 2 | | 0 | | 2 |
| **79** | | Procedure for osteomyelitis of sternum | 0 | | 0 | | 0 | 0 | | 0 | | | 0 | | | 0 | | | 0 | | 2 | | 2 |
| **80** | | Primary repair of nerve | 0 | | 0 | | 0 | 0 | | 0 | | | 0 | | | 0 | | | 1 | | 1 | | 2 |
| **81** | | Transposition of nerve | 0 | | 0 | | 0 | 0 | | 1 | | | 0 | | | 0 | | | 0 | | 1 | | 2 |
| **82** | | Synovectomy of metatarsophalangeal joint | 0 | | 0 | | 0 | 0 | | 1 | | | 0 | | | 0 | | | 0 | | 1 | | 2 |
| **83** | | Procedure for teno-vaginitis | 0 | | 1 | | 0 | 0 | | 0 | | | 0 | | | 0 | | | 0 | | 1 | | 2 |
| **84** | | Procedure for acute osteomyelitis of humerus | 0 | | 0 | | 0 | 0 | | 0 | | | 0 | | | 1 | | | 0 | | 0 | | 1 |
| **85** | | Arthrotomy of shoulder | 0 | | 0 | | 0 | 0 | | 0 | | | 0 | | | 1 | | | 0 | | 0 | | 1 |
| **86** | | Aspiration of joint or other synovial cavity, not elsewhere specified | 1 | | 0 | | 0 | 0 | | 0 | | | 0 | | | 0 | | | 0 | | 0 | | 1 |
| **87** | | Talipes equinovarus, medial release | 0 | | 0 | | 0 | 0 | | 0 | | | 0 | | | 1 | | | 0 | | 0 | | 1 |
| **88** | | Stabilisation of ankle | 0 | | 0 | | 1 | 0 | | 0 | | | 0 | | | 0 | | | 0 | | 0 | | 1 |
| **89** | | Primary repair of extensor tendon of hand | 0 | | 0 | | 0 | 0 | | 0 | | | 0 | | | 0 | | | 1 | | 0 | | 1 |
| **90** | | Scapula, neck or glenoid region, open reduction of | 0 | | 0 | | 0 | 0 | | 0 | | | 0 | | | 0 | | | 1 | | 0 | | 1 |
| **91** | | Transfer of muscle | 0 | | 0 | | 0 | 0 | | 0 | | | 0 | | | 0 | | | 1 | | 0 | | 1 |
|  | **Total** | | **800** | | **838** | | **932** | **851** | | **1073** | | | **1009** | | | **1267** | | | **1298** | | **1324** | | **9392** |
