# Supplementary material for: Randomised Trial Support for Orthopaedic Surgical Procedures
Source: PLoS One. 2014 Jun 13;9(6):e96745. doi: 10.1371/journal.pone.0096745 (PMC4057075; doi:10.1371/journal.pone.0096745)
Supplement: Appendix S2 — Syntax of search strategies for each operative procedure category. (DOCX) [file pone.0096745.s002.docx]

**APPENDIX S2**

**Syntax of search strategies for each operative procedure category**

**Knee arthroscopy**:

1. Arthroscopy/

2. endoscopy/

3. (arthroscop$ or endoscop$).mp.

4. Knee/ or Knee Joint/

5. (knee$ or patellofemoral).mp.

6. 1 or 2 or 3

7. 4 or 5

8. 6 and 7

9. Randomized Controlled Trials as Topic/

10. randomized controlled trial/

11. Random Allocation/

12. Double Blind Method/

13. Single Blind Method/

14. clinical trial/

15. clinical trial, phase i.pt.

16. clinical trial, phase ii.pt.

17. clinical trial, phase iii.pt.

18. clinical trial, phase iv.pt.

19. controlled clinical trial.pt.

20. randomized controlled trial.pt.

21. multicenter study.pt.

22. clinical trial.pt.

23. exp Clinical Trials as topic/

24. or/17-23

25. (clinical adj trial$).tw.

26. ((singl$ or doubl$ or treb$ or tripl$) adj (blind$3 or mask$3)).tw.

27. PLACEBOS/

28. placebo$.tw.

29. randomly allocated.tw.

30. (allocated adj2 random$).tw.

31. or/25-30

32. 24 or 31

33. case report.tw.

34. letter/

35. historical article/

36. or/33-35

37. 32 not 36

38. 8 and 37

**Knee Arthroplasty:**

1. arthroplasty, replacement, knee/ or knee prosthesis/

2. (tka or tka).tw.

3. joint prosthesis/ or arthroplasty/

4. (arthroplast$ or prosthe$ or replac$).tw.

5. 3 or 4

6. Knee/ or Knee Joint/

7. (knee$ or patellofemoral).tw.

8. 6 or 7

9. 5 and 8

10. 1 or 2 or 9

11. Randomized Controlled Trials as Topic/

12. randomized controlled trial/

13. Random Allocation/

14. Double Blind Method/

15. Single Blind Method/

16. clinical trial/

17. clinical trial, phase i.pt.

18. clinical trial, phase ii.pt.

19. clinical trial, phase iii.pt.

20. clinical trial, phase iv.pt.

21. controlled clinical trial.pt.

22. randomized controlled trial.pt.

23. multicenter study.pt.

24. clinical trial.pt.

25. exp Clinical Trials as topic/

26. or/11-25

27. (clinical adj trial$).tw.

28. ((singl$ or doubl$ or treb$ or tripl$) adj (blind$3 or mask$3)).tw.

29. PLACEBOS/

30. placebo$.tw.

31. randomly allocated.tw.

32. (allocated adj2 random$).tw.

33. or/27-32

34. 26 or 33

35. case report.tw.

36. letter/

37. historical article/

38. or/35-37

39. 34 not 38

40. 10 and 39

**Hip Arthroplasty:**

1. arthroplasty, replacement, hip/ or hip prosthesis/

2. (tha or thr).tw.

3. joint prosthesis/ or arthroplasty/

4. (arthroplast$ or prosthe$ or replac$).tw.

5. 3 or 4

6. hip joint/ or hip/

7. (hip$ or femoral head or acetabulum).tw.

8. 6 or 7

9. 5 and 8

10. 1 or 2 or 9

11. Randomized Controlled Trials as Topic/

12. randomized controlled trial/

13. Random Allocation/

14. Double Blind Method/

15. Single Blind Method/

16. clinical trial/

17. clinical trial, phase i.pt.

18. clinical trial, phase ii.pt.

19. clinical trial, phase iii.pt.

20. clinical trial, phase iv.pt.

21. controlled clinical trial.pt.

22. randomized controlled trial.pt.

23. multicenter study.pt.

24. clinical trial.pt.

25. exp Clinical Trials as topic/

26. or/11-25

27. (clinical adj trial$).tw.

28. ((singl$ or doubl$ or treb$ or tripl$) adj (blind$3 or mask$3)).tw.

29. PLACEBOS/

30. placebo$.tw.

31. randomly allocated.tw.

32. (allocated adj2 random$).tw.

33. or/27-32

34. 26 or 33

35. case report.tw.

36. letter/

37. historical article/

38. or/35-37

39. 34 not 38

40. 10 and 39

41. (anaesthe$ or anesthe$ or skin$ or heterotopic$ or intra-articular$ or peri-articular$ or thromboembol$ or thrombo$ or cement$ or uncement$ or ceramic$ or unipol$ or bipol$ or resurfac$)tw.

42. 40 not 41

**Removal / Debridement / Wound Cleaning:**

1. wounds, non penetrating/ or wounds, penetrating/

2. wound$.tw.

3. 1 or 2

4. irrigation/

5. (dress$ or clean$ or irrigat$).tw.

6. 4 or 5

7. 3 and 6

8. (foreign bod$ adj3 remov$).tw.

9. foreign body/

10. 8 or 9

11. debridement/

12. debride$.tw.

13. 11 or 12

14. 7 or 10 or 13

15. Randomized Controlled Trials as Topic/

16. randomized controlled trial/

17. Random Allocation/

18. Double Blind Method/

19. Single Blind Method/

20. clinical trial/

21. clinical trial, phase i.pt.

22. clinical trial, phase ii.pt.

23. clinical trial, phase iii.pt.

24. clinical trial, phase iv.pt.

25. controlled clinical trial.pt.

26. randomized controlled trial.pt.

27. multicenter study.pt.

28. clinical trial.pt.

29. exp Clinical Trials as topic/

30. or/15-29

31. (clinical adj trial$).tw.

32. ((singl$ or doubl$ or treb$ or tripl$) adj (blind$3 or mask$3)).tw.

33. PLACEBOS/

34. placebo$.tw.

35. randomly allocated.tw.

36. (allocated adj2 random$).tw.

37. or/31-36

38. 30 or 37

39. case report.tw.

40. letter/

41. historical article/

42. or/39-41

43. 38 not 42

44. 14 and 43

**Internal Fixation of Proximal Fracture of the Femur:**

1. exp femoral fractures/

2. hip fractures/

3. femoral neck fractures/

4. (trochanteric or subtrochanteric or subcapital or femoral or femur).tw.

5. (fem$ adj3 (neck or head)).tw.

6. nof.tw.

7. (fracture or fractures).tw.

8. 4 or 5 or 6

9. 7 and 8

10. 1 or 2 or 3 or 9

11. Randomized Controlled Trials as Topic/

12. randomized controlled trial/

13. Random Allocation/

14. Double Blind Method/

15. Single Blind Method/

16. clinical trial/

17. clinical trial, phase i.pt.

18. clinical trial, phase ii.pt.

19. clinical trial, phase iii.pt.

20. clinical trial, phase iv.pt.

21. controlled clinical trial.pt.

22. randomized controlled trial.pt.

23. multicenter study.pt.

24. clinical trial.pt.

25. exp Clinical Trials as topic/

26. or/11-25

27. (clinical adj trial$).tw.

28. ((singl$ or doubl$ or treb$ or tripl$) adj (blind$3 or mask$3)).tw.

29. PLACEBOS/

30. placebo$.tw.

31. randomly allocated.tw.

32. (allocated adj2 random$).tw.

33. or/27-32

34. 26 or 33

35. case report.tw.

36. letter/

37. historical article/

38. or/35-37

39. 34 not 38

40. 10 and 39

41. (anaesthe$ or anesthe$ or intra-articular$ or peri-articular$ or thromboembol$ or thrombo$ or cement$ or ceramic$ or unipol$ or bipol$ or resurfac$ or heparin$ or autolog$ or transfusion$ or erythropo$ or propofol$ or morphine$ or tramadol$ or zoledro$ or alendron$ or risedron$ or vitamin$).tw.

42. 40 not 41

**Internal Fixation of Distal Radius Fracture:**

1. Radius Fractures/ or Colles' Fracture/ or Radius/ or wrist injuries/

2. (Barton$ or Smith$ or Colle$ or wrist).tw.

3. (radius or radial).tw.

4. distal.tw.

5. 3 and 4

6. 1 or 2 or 5

7. exp fracture fixation/

8. orthopedics/ or manipulation, orthopedic/ or fractures, bone/

9. exp Orthopedic Fixation Devices/

10. (fixat$ or fracture$ or manipulat$ or realign$ or reposition$ or surg$ or ORIF or reduction).tw.

11. 7 or 8 or 9 or 10

12. 6 and 11

13. Randomized Controlled Trials as Topic/

14. randomized controlled trial/

15. Random Allocation/

16. Double Blind Method/

17. Single Blind Method/

18. clinical trial/

19. clinical trial, phase i.pt.

20. clinical trial, phase ii.pt.

21. clinical trial, phase iii.pt.

22. clinical trial, phase iv.pt.

23. controlled clinical trial.pt.

24. randomized controlled trial.pt.

25. multicenter study.pt.

26. clinical trial.pt.

27. exp Clinical Trials as topic/

28. or/13-27

29. (clinical adj trial$).tw.

30. ((singl$ or doubl$ or treb$ or tripl$) adj (blind$3 or mask$3)).tw.

31. PLACEBOS/

32. placebo$.tw.

33. randomly allocated.tw.

34. (allocated adj2 random$).tw.

35. or/29-34

36. 28 or 35

37. case report.tw.

38. letter/

39. historical article/

40. or/37-39

41. 36 not 40

42. 12 and 41

43. (anaesthe$ or anesthe$ or intra-articular$ or peri-articular$ or thromboembol$ or thrombo$ or heparin$ or autolog$ or transfusion$ or erythropo$ or propofol$ or morphine$ or tramadol$ or collect$ or colleg$).tw.

44. 42 not 43

**Removal of Implants:**

1. orthopedic fixation devices/ or bone nails/ or bone plates/ or bone screws/ or bone wires/ or external fixators/ or internal fixators/ or suture anchors/

2. surgical fixation devices/

3. (nail$ or plate$ or screw$ or wire$ or fixat$ or rod$ or K-wires$ or prosthe$ or implant$).tw.

4. hip prosthesis/ or knee prosthesis/

5. 1 or 2 or 3 or 4

6. Device Removal/

7. (remov$ or extract$ or retract$).tw.

8. 6 or 7

9. 5 and 8

10. Randomized Controlled Trials as Topic/

11. randomized controlled trial/

12. Random Allocation/

13. Double Blind Method/

14. Single Blind Method/

15. clinical trial/

16. clinical trial, phase i.pt.

17. clinical trial, phase ii.pt.

18. clinical trial, phase iii.pt.

19. clinical trial, phase iv.pt.

20. controlled clinical trial.pt.

21. randomized controlled trial.pt.

22. multicenter study.pt.

23. clinical trial.pt.

24. exp Clinical Trials as topic/

25. or/10-24

26. (clinical adj trial$).tw.

27. ((singl$ or doubl$ or treb$ or tripl$) adj (blind$3 or mask$3)).tw.

28. PLACEBOS/

29. placebo$.tw.

30. randomly allocated.tw.

31. (allocated adj2 random$).tw.

32. or/26-31

33. 25 or 32

34. case report.tw.

35. letter/

36. historical article/

37. or/34-36

38. 33 not 37

39. 9 and 38

40. (mandib$ or maxill$ or tooth$ or teeth$ or molar$ or oral$ or dental$ or dentist$ or orthodont$ or stent$ or cardia$ or myocardi$ or cardiopulm$ or catheter$ or norplant$ or pregnan$ or prostate$ or platelet or onychomyc$ or intraocul$ or ocula$ or cornea$ or cataract$).tw.

41. 39 not 40

**Ankle Fracture Fixation:**

1. Ankle/ or Ankle Joint/ or Ankle Injuries/

2. (ankle$ or talocrural or foot or calcane$).tw.

3. 1 or 2

4. exp Fracture Fixation/

5. Orthopedics/

6. Manipulation, Orthopedic/

7. exp Orthopedic Fixation Devices/

8. exp Fractures, Bone/

9. (fixat$ or manipulat$ or realign$ or reposition$ or surg$ or ORIF).tw.

10. 4 or 5 or 6 or 7 or 8 or 9

11. 3 and 10

12. Randomized Controlled Trials as Topic/

13. randomized controlled trial/

14. Random Allocation/

15. Double Blind Method/

16. Single Blind Method/

17. clinical trial/

18. clinical trial, phase i.pt.

19. clinical trial, phase ii.pt.

20. clinical trial, phase iii.pt.

21. clinical trial, phase iv.pt.

22. controlled clinical trial.pt.

23. randomized controlled trial.pt.

24. multicenter study.pt.

25. clinical trial.pt.

26. exp Clinical Trials as topic/

27. or/12-26

28. (clinical adj trial$).tw.

29. ((singl$ or doubl$ or treb$ or tripl$) adj (blind$3 or mask$3)).tw.

30. PLACEBOS/

31. placebo$.tw.

32. randomly allocated.tw.

33. (allocated adj2 random$).tw.

34. or/28-33

35. 27 or 34

36. case report.tw.

37. letter/

38. historical article/

39. or/36-38

40. 35 not 39

41. 11 and 40

**Acromioplasty / Repair of Rotator Cuff:**

1. shoulder joint/ or rotator cuff/

2. (shoulder$ or glenohumeral or rotator cuff).tw.

3. shoulder impingement syndrome/

4. 1 or 2 or 3

5. (acromioplasty or repair$).tw.

6. 4 and 5

7. Randomized Controlled Trials as Topic/

8. randomized controlled trial/

9. Random Allocation/

10. Double Blind Method/

11. Single Blind Method/

12. clinical trial/

13. clinical trial, phase i.pt.

14. clinical trial, phase ii.pt.

15. clinical trial, phase iii.pt.

16. clinical trial, phase iv.pt.

17. controlled clinical trial.pt.

18. randomized controlled trial.pt.

19. multicenter study.pt.

20. clinical trial.pt.

21. exp Clinical Trials as topic/

22. or/7-21

23. (clinical adj trial$).tw.

24. ((singl$ or doubl$ or treb$ or tripl$) adj (blind$3 or mask$3)).tw.

25. PLACEBOS/

26. placebo$.tw.

27. randomly allocated.tw.

28. (allocated adj2 random$).tw.

29. or/23-28

30. 22 or 29

31. case report.tw.

32. letter/

33. historical article/

34. or/31-33

35. 30 not 34

36. 6 and 35

**Shoulder Arthroscopy:**

1. Arthroscopy/

2. endoscopy/

3. (arthroscop$ or endoscop$).mp.

4. Shoulder Joint/ or Shoulder/

5. (shoulder$ or glenohumeral).mp.

6. 1 or 2 or 3

7. 4 or 5

8. 6 and 7

9. Randomized Controlled Trials as Topic/

10. randomized controlled trial/

11. Random Allocation/

12. Double Blind Method/

13. Single Blind Method/

14. clinical trial/

15. clinical trial, phase i.pt.

16. clinical trial, phase ii.pt.

17. clinical trial, phase iii.pt.

18. clinical trial, phase iv.pt.

19. controlled clinical trial.pt.

20. randomized controlled trial.pt.

21. multicenter study.pt.

22. clinical trial.pt.

23. exp Clinical Trials as topic/

24. or/9-23

25. (clinical adj trial$).tw.

26. ((singl$ or doubl$ or treb$ or tripl$) adj (blind$3 or mask$3)).tw.

27. PLACEBOS/

28. placebo$.tw.

29. randomly allocated.tw.

30. (allocated adj2 random$).tw.

31. or/25-30

32. 24 or 31

33. case report.tw.

34. letter/

35. historical article/

36. or/33-35

37. 32 not 36

38. 8 and 37

**Open Reduction of Fracture of Shaft of Tibia with Internal Fixation:**

1. Tibial fractures/ or Tibia/

2. (tibia or tibial or shin).tw.

3. shaft.tw.

4. 2 and 3

5. 1 or 4

6. exp fracture fixation/

7. orthopedics/ or manipulation, orthopedic/ or fractures, bone/

8. exp Orthopedic Fixation Devices/

9. (fixat$ or fracture$ or manipulat$ or realign$ or reposition$ or surg$ or ORIF or reduction).tw.

10. 6 or 7 or 8 or 9

11. 5 and 10

12. Randomized Controlled Trials as Topic/

13. randomized controlled trial/

14. Random Allocation/

15. Double Blind Method/

16. Single Blind Method/

17. clinical trial/

18. clinical trial, phase i.pt.

19. clinical trial, phase ii.pt.

20. clinical trial, phase iii.pt.

21. clinical trial, phase iv.pt.

22. controlled clinical trial.pt.

23. randomized controlled trial.pt.

24. multicenter study.pt.

25. clinical trial.pt.

26. exp Clinical Trials as topic/

27. or/12-26

28. (clinical adj trial$).tw.

29. ((singl$ or doubl$ or treb$ or tripl$) adj (blind$3 or mask$3)).tw.

30. PLACEBOS/

31. placebo$.tw.

32. randomly allocated.tw.

33. (allocated adj2 random$).tw.

34. or/28-33

35. 27 or 34

36. case report.tw.

37. letter/

38. historical article/

39. or/36-38

40. 35 not 39

41. 11 and 40

**Osteotomy:**

1. Osteotomy/

2. (realign$ or osteotom$).tw.

3. 1 or 2

4. Randomized Controlled Trials as Topic/

5. randomized controlled trial/

6. Random Allocation/

7. Double Blind Method/

8. Single Blind Method/

9. clinical trial/

10. clinical trial, phase i.pt.

11. clinical trial, phase ii.pt.

12. clinical trial, phase iii.pt.

13. clinical trial, phase iv.pt.

14. controlled clinical trial.pt.

15. randomized controlled trial.pt.

16. multicenter study.pt.

17. clinical trial.pt.

18. exp Clinical Trials as topic/

19. or/4-18

20. (clinical adj trial$).tw.

21. ((singl$ or doubl$ or treb$ or tripl$) adj (blind$3 or mask$3)).tw.

22. PLACEBOS/

23. placebo$.tw.

24. randomly allocated.tw.

25. (allocated adj2 random$).tw.

26. or/20-25

27. 19 or 26

28. case report.tw.

29. letter/

30. historical article/

31. or/28-30

32. 27 not 31

33. 3 and 32

**Open Reduction of Joint Dislocation (Shoulder, Acromioclavicular & Patella):**

Shoulder:

1. shoulder joint/ or shoulder/

2. (shoulder$ or glenohumeral).tw.

3. 1 or 2

4. dislocations/ or shoulder dislocation/

5. dislocat$.tw.

6. 4 or 5

7. 3 and 6

8. Randomized Controlled Trials as Topic/

9. randomized controlled trial/

10. Random Allocation/

11. Double Blind Method/

12. Single Blind Method/

13. clinical trial/

14. clinical trial, phase i.pt.

15. clinical trial, phase ii.pt.

16. clinical trial, phase iii.pt.

17. clinical trial, phase iv.pt.

18. controlled clinical trial.pt.

19. randomized controlled trial.pt.

20. multicenter study.pt.

21. clinical trial.pt.

22. exp Clinical Trials as topic/

23. or/8-22

24. (clinical adj trial$).tw.

25. ((singl$ or doubl$ or treb$ or tripl$) adj (blind$3 or mask$3)).tw.

26. PLACEBOS/

27. placebo$.tw.

28. randomly allocated.tw.

29. (allocated adj2 random$).tw.

30. or/24-29

31. 23 or 30

32. case report.tw.

33. letter/

34. historical article/

35. or/32-34

36. 31 not 35

37. 7 and 36

Acromioclavicular:

1. clavicle/

2. (clavicle$ or collar bone$ or sternoclavicular or acromioclavicular or sterno-clavicular or acromio-clavicular).tw.

3. 1 or 2

4. Dislocations/

5. dislocat$.tw.

6. 4 or 5

7. 3 and 6

8. Randomized Controlled Trials as Topic/

9. randomized controlled trial/

10. Random Allocation/

11. Double Blind Method/

12. Single Blind Method/

13. clinical trial/

14. clinical trial, phase i.pt.

15. clinical trial, phase ii.pt.

16. clinical trial, phase iii.pt.

17. clinical trial, phase iv.pt.

18. controlled clinical trial.pt.

19. randomized controlled trial.pt.

20. multicenter study.pt.

21. clinical trial.pt.

22. exp Clinical Trials as topic/

23. or/8-22

24. (clinical adj trial$).tw.

25. ((singl$ or doubl$ or treb$ or tripl$) adj (blind$3 or mask$3)).tw.

26. PLACEBOS/

27. placebo$.tw.

28. randomly allocated.tw.

29. (allocated adj2 random$).tw.

30. or/24-29

31. 23 or 30

32. case report.tw.

33. letter/

34. historical article/

35. or/32-34

36. 31 not 35

37. 7 and 36

Patella:

1. patella/

2. (patella or knee cap or kneepan).tw.

3. 1 or 2

4. dislocations/ or patellar dislocation/

5. dislocat$.tw.

6. 4 or 5

7. 3 and 6

8. Randomized Controlled Trials as Topic/

9. randomized controlled trial/

10. Random Allocation/

11. Double Blind Method/

12. Single Blind Method/

13. clinical trial/

14. clinical trial, phase i.pt.

15. clinical trial, phase ii.pt.

16. clinical trial, phase iii.pt.

17. clinical trial, phase iv.pt.

18. controlled clinical trial.pt.

19. randomized controlled trial.pt.

20. multicenter study.pt.

21. clinical trial.pt.

22. exp Clinical Trials as topic/

23. or/8-22

24. (clinical adj trial$).tw.

25. ((singl$ or doubl$ or treb$ or tripl$) adj (blind$3 or mask$3)).tw.

26. PLACEBOS/

27. placebo$.tw.

28. randomly allocated.tw.

29. (allocated adj2 random$).tw.

30. or/24-29

31. 23 or 30

32. case report.tw.

33. letter/

34. historical article/

35. or/32-34

36. 31 not 35

37. 7 and 36

**Knee, Repair of Cruciate Ligament:**

1. anterior cruciate ligament/ or posterior cruciate ligament/

2. (cruciate or ACL or PCL).tw.

3. 1 or 2

4. (reconstruct$ or repair$ or surg$ or operat$).tw.

5. 3 and 4

6. Randomized Controlled Trials as Topic/

7. randomized controlled trial/

8. Random Allocation/

9. Double Blind Method/

10. Single Blind Method/

11. clinical trial/

12. clinical trial, phase i.pt.

13. clinical trial, phase ii.pt.

14. clinical trial, phase iii.pt.

15. clinical trial, phase iv.pt.

16. controlled clinical trial.pt.

17. randomized controlled trial.pt.

18. multicenter study.pt.

19. clinical trial.pt.

20. exp Clinical Trials as topic/

21. or/6-20

22. (clinical adj trial$).tw.

23. ((singl$ or doubl$ or treb$ or tripl$) adj (blind$3 or mask$3)).tw.

24. PLACEBOS/

25. placebo$.tw.

26. randomly allocated.tw.

27. (allocated adj2 random$).tw.

28. or/22-27

29. 21 or 28

30. case report.tw.

31. letter/

32. historical article/

33. or/30-32

34. 29 not 33

35. 5 and 34

**Tibia, Plateau of, Medial or Lateral fracture, Open Reduction with Internal Fixation:**

1. Tibial fractures/ or Tibia/

2. (tibia or tibial or shin).tw.

3. plateau.tw.

4. 2 and 3

5. 1 or 4

6. exp fracture fixation/

7. orthopedics/ or manipulation, orthopedic/ or fractures, bone/

8. exp orthopedic fixation devices/

9. (fixat$ or fracture$ or manipulat$ or realign$ or reposition$ or surg$ or ORIF or reduction).tw.

10. 6 or 7 or 8 or 9

11. 5 and 10

12. Randomized Controlled Trials as Topic/

13. randomized controlled trial/

14. Random Allocation/

15. Double Blind Method/

16. Single Blind Method/

17. clinical trial/

18. clinical trial, phase i.pt.

19. clinical trial, phase ii.pt.

20. clinical trial, phase iii.pt.

21. clinical trial, phase iv.pt.

22. controlled clinical trial.pt.

23. randomized controlled trial.pt.

24. multicenter study.pt.

25. clinical trial.pt.

26. exp Clinical Trials as topic/

27. or/12-26

28. (clinical adj trial$).tw.

29. ((singl$ or doubl$ or treb$ or tripl$) adj (blind$3 or mask$3)).tw.

30. PLACEBOS/

31. placebo$.tw.

32. randomly allocated.tw.

33. (allocated adj2 random$).tw.

34. or/28-33

35. 27 or 34

36. case report.tw.

37. letter/

38. historical article/

39. or/36-38

40. 35 not 39

41. 11 and 40

**Repair of Achilles Tendon Rupture:**

1. Achilles Tendon/

2. achilles.tw.

3. 1 or 2

4. Randomized Controlled Trials as Topic/

5. randomized controlled trial/

6. Random Allocation/

7. Double Blind Method/

8. Single Blind Method/

9. clinical trial/

10. clinical trial, phase i.pt.

11. clinical trial, phase ii.pt.

12. clinical trial, phase iii.pt.

13. clinical trial, phase iv.pt.

14. controlled clinical trial.pt.

15. randomized controlled trial.pt.

16. multicenter study.pt.

17. clinical trial.pt.

18. exp Clinical Trials as topic/

19. or/4-18

20. (clinical adj trial$).tw.

21. ((singl$ or doubl$ or treb$ or tripl$) adj (blind$3 or mask$3)).tw.

22. PLACEBOS/

23. placebo$.tw.

24. randomly allocated.tw.

25. (allocated adj2 random$).tw.

26. or/20-25

27. 19 or 26

28. case report.tw.

29. letter/

30. historical article/

31. or/28-30

32. 27 not 31

33. 3 and 32

**Humerus, Distal, Treatment of Fracture by Open Reduction with Internal Fixation:**

1. Humeral Fractures/

2. (humerus or humeral).tw.

3. exp Fracture Fixation/

4. exp Orthopedic Fixation Devices/

5. (fixat$ or fracture$ or manipulat$ or realign$ or reposition$ or surg$ or ORIF or reduction).tw.

6. orthopedics/ or manipulation, orthopedic/ or fractures, bone/

7. 3 or 4 or 5 or 6

8. 2 and 7

9. 1 or 8

10. Randomized Controlled Trials as Topic/

11. randomized controlled trial/

12. Random Allocation/

13. Double Blind Method/

14. Single Blind Method/

15. clinical trial/

16. clinical trial, phase i.pt.

17. clinical trial, phase ii.pt.

18. clinical trial, phase iii.pt.

19. clinical trial, phase iv.pt.

20. controlled clinical trial.pt.

21. randomized controlled trial.pt.

22. multicenter study.pt.

23. clinical trial.pt.

24. exp Clinical Trials as topic/

25. or/10-24

26. (clinical adj trial$).tw.

27. ((singl$ or doubl$ or treb$ or tripl$) adj (blind$3 or mask$3)).tw.

28. PLACEBOS/

29. placebo$.tw.

30. randomly allocated.tw.

31. (allocated adj2 random$).tw.

32. or/26-31

33. 25 or 32

34. case report.tw.

35. letter/

36. historical article/

37. or/34-36

38. 33 not 37

39. 9 and 38

**Olecranon, Treatment of Fracture by Open Reduction with Internal Fixation:**

1. Olecranon Process/ or Elbow Joint/

2. (olecranon or elbow$).tw.

3. 1 or 2

4. exp Fracture Fixation/

5. orthopedics/ or manipulation, orthopedic/ or fractures, bone/

6. exp Orthopedic Fixation Devices/

7. (fixat$ or fracture$ or manipulat$ or realign$ or reposition$ or surg$ or ORIF or reduction).tw.

8. 4 or 5 or 6 or 7

9. 3 and 8

10. Randomized Controlled Trials as Topic/

11. randomized controlled trial/

12. Random Allocation/

13. Double Blind Method/

14. Single Blind Method/

15. clinical trial/

16. clinical trial, phase i.pt.

17. clinical trial, phase ii.pt.

18. clinical trial, phase iii.pt.

19. clinical trial, phase iv.pt.

20. controlled clinical trial.pt.

21. randomized controlled trial.pt.

22. multicenter study.pt.

23. clinical trial.pt.

24. exp Clinical Trials as topic/

25. or/10-24

26. (clinical adj trial$).tw.

27. ((singl$ or doubl$ or treb$ or tripl$) adj (blind$3 or mask$3)).tw.

28. PLACEBOS/

29. placebo$.tw.

30. randomly allocated.tw.

31. (allocated adj2 random$).tw.

32. or/26-31

33. 25 or 32

34. case report.tw.

35. letter/

36. historical article/

37. or/34-36

38. 33 not 37

39. 9 and 38

**Arthroscopy of Ankle:**

1. Arthroscopy/ or Endoscopy/

2. (arthroscop$ or endoscop$).tw.

3. Ankle/ or Ankle Joint/

4. (ankle$ or foot).tw.

5. 1 or 2

6. 3 or 4

7. 5 and 6

8. Randomized Controlled Trials as Topic/

9. randomized controlled trial/

10. Random Allocation/

11. Double Blind Method/

12. Single Blind Method/

13. clinical trial/

14. clinical trial, phase i.pt.

15. clinical trial, phase ii.pt.

16. clinical trial, phase iii.pt.

17. clinical trial, phase iv.pt.

18. controlled clinical trial.pt.

19. randomized controlled trial.pt.

20. multicenter study.pt.

21. clinical trial.pt.

22. exp Clinical Trials as topic/

23. or/8-22

24. (clinical adj trial$).tw.

25. ((singl$ or doubl$ or treb$ or tripl$) adj (blind$3 or mask$3)).tw.

26. PLACEBOS/

27. placebo$.tw.

28. randomly allocated.tw.

29. (allocated adj2 random$).tw.

30. or/24-29

31. 23 or 30

32. case report.tw.

33. letter/

34. historical article/

35. or/32-34

36. 31 not 35

37. 7 and 36

**Joint Arthrodesis:**

1. arthrodesis/

2. ankylosis/

3. (ankylos$ or syndesis or arthrodesis or bone transplant$ or bone regenerat$).tw.

4. 1 or 2 or 3

5. Randomized Controlled Trials as Topic/

6. randomized controlled trial/

7. Random Allocation/

8. Double Blind Method/

9. Single Blind Method/

10. clinical trial/

11. clinical trial, phase i.pt.

12. clinical trial, phase ii.pt.

13. clinical trial, phase iii.pt.

14. clinical trial, phase iv.pt.

15. controlled clinical trial.pt.

16. randomized controlled trial.pt.

17. multicenter study.pt.

18. clinical trial.pt.

19. exp Clinical Trials as topic/

20. or/5-19

21. (clinical adj trial$).tw.

22. ((singl$ or doubl$ or treb$ or tripl$) adj (blind$3 or mask$3)).tw.

23. PLACEBOS/

24. placebo$.tw.

25. randomly allocated.tw.

26. (allocated adj2 random$).tw.

27. or/21-26

28. 20 or 27

29. case report.tw.

30. letter/

31. historical article/

32. or/29-31

33. 28 not 32

34. 4 and 33

**Abscess Drainage:**

1. abscess/ or pus/

2. (absces$ or pus or collection).tw.

3. 1 or 2

4. drainage/

5. (draina$ or incisio$ or irrigat$).tw.

6. 4 or 5

7. 3 and 6

8. Randomized Controlled Trials as Topic/

9. randomized controlled trial/

10. Random Allocation/

11. Double Blind Method/

12. Single Blind Method/

13. clinical trial/

14. clinical trial, phase i.pt.

15. clinical trial, phase ii.pt.

16. clinical trial, phase iii.pt.

17. clinical trial, phase iv.pt.

18. controlled clinical trial.pt.

19. randomized controlled trial.pt.

20. multicenter study.pt.

21. clinical trial.pt.

22. exp Clinical Trials as topic/

23. or/8-22

24. (clinical adj trial$).tw.

25. ((singl$ or doubl$ or treb$ or tripl$) adj (blind$3 or mask$3)).tw.

26. PLACEBOS/

27. placebo$.tw.

28. randomly allocated.tw.

29. (allocated adj2 random$).tw.

30. or/24-29

31. 23 or 30

32. case report.tw.

33. letter/

34. historical article/

35. or/32-34

36. 31 not 35

37. 7 and 36

**Clavicle, Treatment of Fracture, Open reduction with Internal Fixation:**

1. Clavicle/

2. (clavicle$ or collar bone$ or sternoclavicular or acromioclavicular or sterno-clavicular or acromio-clavicular).tw.

3. 1 or 2

4. exp Fracture Fixation/

5. orthopedics/ or manipulation, orthopedic/ or fractures, bone/

6. exp Orthopedic Fixation Devices/

7. (fixat$ or fracture$ or manipulat$ or realign$ or reposition$ or surg$ or ORIF or reduction).tw.

8. 4 or 5 or 6 or 7

9. 3 and 8

10. Randomized Controlled Trials as Topic/

11. randomized controlled trial/

12. Random Allocation/

13. Double Blind Method/

14. Single Blind Method/

15. clinical trial/

16. clinical trial, phase i.pt.

17. clinical trial, phase ii.pt.

18. clinical trial, phase iii.pt.

19. clinical trial, phase iv.pt.

20. controlled clinical trial.pt.

21. randomized controlled trial.pt.

22. multicenter study.pt.

23. clinical trial.pt.

24. exp Clinical Trials as topic/

25. or/10-24

26. (clinical adj trial$).tw.

27. ((singl$ or doubl$ or treb$ or tripl$) adj (blind$3 or mask$3)).tw.

28. PLACEBOS/

29. placebo$.tw.

30. randomly allocated.tw.

31. (allocated adj2 random$).tw.

32. or/26-31

33. 25 or 32

34. case report.tw.

35. letter/

36. historical article/

37. or/34-36

38. 33 not 37

39. 9 and 38

**Patella, Treatment Fracture, by Open Reduction with Internal Fixation:**

1. Patella/

2. (patella or knee cap or kneepan).tw.

3. 1 or 2

4. exp Fracture Fixation/

5. orthopedics/ or manipulation, orthopedic/ or fractures, bone/

6. exp Orthopedic Fixation Devices/

7. (fixat$ or fracture$ or manipulat$ or realign$ or reposition$ or surg$ or ORIF or reduction).tw.

8. 4 or 5 or 6 or 7

9. 3 and 8

10. Randomized Controlled Trials as Topic/

11. randomized controlled trial/

12. Random Allocation/

13. Double Blind Method/

14. Single Blind Method/

15. clinical trial/

16. clinical trial, phase i.pt.

17. clinical trial, phase ii.pt.

18. clinical trial, phase iii.pt.

19. clinical trial, phase iv.pt.

20. controlled clinical trial.pt.

21. randomized controlled trial.pt.

22. multicenter study.pt.

23. clinical trial.pt.

24. exp Clinical Trials as topic/

25. or/10-24

26. (clinical adj trial$).tw.

27. ((singl$ or doubl$ or treb$ or tripl$) adj (blind$3 or mask$3)).tw.

28. PLACEBOS/

29. placebo$.tw.

30. randomly allocated.tw.

31. (allocated adj2 random$).tw.

32. or/26-31

33. 25 or 32

34. case report.tw.

35. letter/

36. historical article/

37. or/34-36

38. 33 not 37

39. 9 and 38

**Humerus, Proximal, Treatment of Fracture by Open Reduction with Internal Fixation:**

1. Humeral Fractures/

2. (humerus or humeral).tw.

3. exp Fracture Fixation/

4. exp Orthopedic Fixation Devices/

5. (fixat$ or fracture$ or manipulat$ or realign$ or reposition$ or surg$ or ORIF or reduction).tw.

6. orthopedics/ or manipulation, orthopedic/ or fractures, bone/

7. 3 or 4 or 5 or 6

8. 2 and 7

9. 1 or 8

10. Randomized Controlled Trials as Topic/

11. randomized controlled trial/

12. Random Allocation/

13. Double Blind Method/

14. Single Blind Method/

15. clinical trial/

16. clinical trial, phase i.pt.

17. clinical trial, phase ii.pt.

18. clinical trial, phase iii.pt.

19. clinical trial, phase iv.pt.

20. controlled clinical trial.pt.

21. randomized controlled trial.pt.

22. multicenter study.pt.

23. clinical trial.pt.

24. exp Clinical Trials as topic/

25. or/10-24

26. (clinical adj trial$).tw.

27. ((singl$ or doubl$ or treb$ or tripl$) adj (blind$3 or mask$3)).tw.

28. PLACEBOS/

29. placebo$.tw.

30. randomly allocated.tw.

31. (allocated adj2 random$).tw.

32. or/26-31

33. 25 or 32

34. case report.tw.

35. letter/

36. historical article/

37. or/34-36

38. 33 not 37

39. 9 and 38

**Amputation:**

1. amputation/

2. amputat$.tw.

3. 1 or 2

4. Randomized Controlled Trials as Topic/

5. randomized controlled trial/

6. Random Allocation/

7. Double Blind Method/

8. Single Blind Method/

9. clinical trial/

10. clinical trial, phase i.pt.

11. clinical trial, phase ii.pt.

12. clinical trial, phase iii.pt.

13. clinical trial, phase iv.pt.

14. controlled clinical trial.pt.

15. randomized controlled trial.pt.

16. multicenter study.pt.

17. clinical trial.pt.

18. exp Clinical Trials as topic/

19. or/4-18

20. (clinical adj trial$).tw.

21. ((singl$ or doubl$ or treb$ or tripl$) adj (blind$3 or mask$3)).tw.

22. PLACEBOS/

23. placebo$.tw.

24. randomly allocated.tw.

25. (allocated adj2 random$).tw.

26. or/20-25

27. 19 or 26

28. case report.tw.

29. letter/

30. historical article/

31. or/28-30

32. 27 not 31

33. 3 and 32

34. (analges$ or anaesth$ or anesth$).tw.

35. 33 not 34

**Foot (not Talus or Calcaneus) Fracture Fixation:**

1. Foot/ or Foot fracture/

2. (foot or feet or lisfranc$ or midfoot$).tw.

3. 1 or 2

4. exp Fracture Fixation/

5. Orthopedics/

6. Manipulation, Orthopedic/

7. exp Orthopedic Fixation Devices/

8. exp Fractures, Bone/

9. (fixat$ or manipulat$ or realign$ or reposition$ or surg$ or ORIF).tw.

10. 4 or 5 or 6 or 7 or 8 or 9

11. 3 and 10

12. Randomized Controlled Trials as Topic/

13. randomized controlled trial/

14. Random Allocation/

15. Double Blind Method/

16. Single Blind Method/

17. clinical trial/

18. clinical trial, phase i.pt.

19. clinical trial, phase ii.pt.

20. clinical trial, phase iii.pt.

21. clinical trial, phase iv.pt.

22. controlled clinical trial.pt.

23. randomized controlled trial.pt.

24. multicenter study.pt.

25. clinical trial.pt.

26. exp Clinical Trials as topic/

27. or/12-26

28. (clinical adj trial$).tw.

29. ((singl$ or doubl$ or treb$ or tripl$) adj (blind$3 or mask$3)).tw.

30. PLACEBOS/

31. placebo$.tw.

32. randomly allocated.tw.

33. (allocated adj2 random$).tw.

34. or/28-33

35. 27 or 34

36. case report.tw.

37. letter/

38. historical article/

39. or/36-38

40. 35 not 39

41. 11 and 40

42. (calcan$ or talus$ or talar$ or ulcer$ or diabet$ or football$ or analges$ or anaesth$ or anesth$ or vascular$ or pneumat$).tw.

43. 41 not 42

**Acetabulum, Treatment of Fracture by Open Reduction with Internal Fixation:**

1. acetabulum/ or hip joint/

2. (acetabulum or acetabular or hip joint$).tw.

3. 1 or 2

4. exp Fracture Fixation/

5. orthopedics/ or manipulation, orthopedic/ or fractures, bone/

6. exp Orthopedic Fixation Devices/

7. (fixat$ or fracture$ or manipulat$ or realign$ or reposition$ or surg$ or ORIF or reduction).tw.

8. 4 or 5 or 6 or 7

9. 3 and 8

10. Randomized Controlled Trials as Topic/

11. randomized controlled trial/

12. Random Allocation/

13. Double Blind Method/

14. Single Blind Method/

15. clinical trial/

16. clinical trial, phase i.pt.

17. clinical trial, phase ii.pt.

18. clinical trial, phase iii.pt.

19. clinical trial, phase iv.pt.

20. controlled clinical trial.pt.

21. randomized controlled trial.pt.

22. multicenter study.pt.

23. clinical trial.pt.

24. exp Clinical Trials as topic/

25. or/10-24

26. (clinical adj trial$).tw.

27. ((singl$ or doubl$ or treb$ or tripl$) adj (blind$3 or mask$3)).tw.

28. PLACEBOS/

29. placebo$.tw.

30. randomly allocated.tw.

31. (allocated adj2 random$).tw.

32. or/26-31

33. 25 or 32

34. case report.tw.

35. letter/

36. historical article/

37. or/34-36

38. 33 not 37

39. 9 and 38

**Excision of Ganglion:**

1. Ganglion/

2. ganglion.tw.

3. 1 or 2

4. (excis$ and gangiion).tw.

5. 3 or 4

6. Randomized Controlled Trials as Topic/

7. randomized controlled trial/

8. Random Allocation/

9. Double Blind Method/

10. Single Blind Method/

11. clinical trial/

12. clinical trial, phase i.pt.

13. clinical trial, phase ii.pt.

14. clinical trial, phase iii.pt.

15. clinical trial, phase iv.pt.

16. controlled clinical trial.pt.

17. randomized controlled trial.pt.

18. multicenter study.pt.

19. clinical trial.pt.

20. exp Clinical Trials as topic/

21. or/6-20

22. (clinical adj trial$).tw.

23. ((singl$ or doubl$ or treb$ or tripl$) adj (blind$3 or mask$3)).tw.

24. PLACEBOS/

25. placebo$.tw.

26. randomly allocated.tw.

27. (allocated adj2 random$).tw.

28. or/22-27

29. 21 or 28

30. case report.tw.

31. letter/

32. historical article/

33. or/30-32

34. 29 not 33

35. 5 and 34

**Wedge Resection of Ingrown Toenail:**

1. Nails, Ingrown/ or Onychocryptosis/

2. (ingrow$ or onychocryptosis or unguis incarnatus or toenail$).tw.

3. 1 or 2

4. (wedge resection or lateral onychoplasty).tw.

5. 3 or 4

6. Randomized Controlled Trials as Topic/

7. randomized controlled trial/

8. Random Allocation/

9. Double Blind Method/

10. Single Blind Method/

11. clinical trial/

12. clinical trial, phase i.pt.

13. clinical trial, phase ii.pt.

14. clinical trial, phase iii.pt.

15. clinical trial, phase iv.pt.

16. controlled clinical trial.pt.

17. randomized controlled trial.pt.

18. multicenter study.pt.

19. clinical trial.pt.

20. exp Clinical Trials as topic/

21. or/6-20

22. (clinical adj trial$).tw.

23. ((singl$ or doubl$ or treb$ or tripl$) adj (blind$3 or mask$3)).tw.

24. PLACEBOS/

25. placebo$.tw.

26. randomly allocated.tw.

27. (allocated adj2 random$).tw.

28. or/22-27

29. 21 or 28

30. case report.tw.

31. letter/

32. historical article/

33. or/30-32

34. 29 not 33

35. 5 and 34

**Release of Carpal Tunnel:**

1. Syndrome, carpal tunnel/

2. carpal tunnel syndrome.tw.

3. 1 or 2

4. Randomized Controlled Trials as Topic/

5. randomized controlled trial/

6. Random Allocation/

7. Double Blind Method/

8. Single Blind Method/

9. clinical trial/

10. clinical trial, phase i.pt.

11. clinical trial, phase ii.pt.

12. clinical trial, phase iii.pt.

13. clinical trial, phase iv.pt.

14. controlled clinical trial.pt.

15. randomized controlled trial.pt.

16. multicenter study.pt.

17. clinical trial.pt.

18. exp Clinical Trials as topic/

19. or/4-18

20. (clinical adj trial$).tw.

21. ((singl$ or doubl$ or treb$ or tripl$) adj (blind$3 or mask$3)).tw.

22. PLACEBOS/

23. placebo$.tw.

24. randomly allocated.tw.

25. (allocated adj2 random$).tw.

26. or/20-25

27. 19 or 26

28. case report.tw.

29. letter/

30. historical article/

31. or/28-30

32. 27 not 31

33. 3 and 32

**Shoulder Arthroplasty:**

1. arthroplasty, replacement, shoulder/ or shoulder prosthesis/

2. (tsa or tsr).tw.

3. joint prosthesis/ or arthroplasty/

4. (arthroplast$ or prosthe$ or replac$).tw.

5. 3 or 4

6. shoulder joint/ or shoulder/

7. (shoulder$ or glenohumeral).tw.

8. 6 or 7

9. 5 and 8

10. 1 or 2 or 9

11. Randomized Controlled Trials as Topic/

12. randomized controlled trial/

13. Random Allocation/

14. Double Blind Method/

15. Single Blind Method/

16. clinical trial/

17. clinical trial, phase i.pt.

18. clinical trial, phase ii.pt.

19. clinical trial, phase iii.pt.

20. clinical trial, phase iv.pt.

21. controlled clinical trial.pt.

22. randomized controlled trial.pt.

23. multicenter study.pt.

24. clinical trial.pt.

25. exp Clinical Trials as topic/

26. or/11-25

27. (clinical adj trial$).tw.

28. ((singl$ or doubl$ or treb$ or tripl$) adj (blind$3 or mask$3)).tw.

29. PLACEBOS/

30. placebo$.tw.

31. randomly allocated.tw.

32. (allocated adj2 random$).tw.

33. or/27-32

34. 26 or 33

35. case report.tw.

36. letter/

37. historical article/

38. or/35-37

39. 34 not 38

40. 10 and 39

41. (anaesthe$ or anesthe$ or skin$ or heterotopic$ or intra-articular$ or peri-articular$ or thromboembol$ or thrombo$ or cement$ or uncement$ or ceramic$ or unipol$ or bipol$ or resurfac$ or heparin$ or autolog$ or transfusion$ or erythropo$ or hormone$ or smok$ or propofol$ or morphine$ or tramadol$).tw.

42. 40 not 41

**Fasciotomy:**

1. Syndrome, compartment/

2. compartment syndrome.tw.

3. 1 or 2

4. (Fasciotomy or fasciectomy).tw.

5. 3 or 4

6. Randomized Controlled Trials as Topic/

7. randomized controlled trial/

8. Random Allocation/

9. Double Blind Method/

10. Single Blind Method/

11. clinical trial/

12. clinical trial, phase i.pt.

13. clinical trial, phase ii.pt.

14. clinical trial, phase iii.pt.

15. clinical trial, phase iv.pt.

16. controlled clinical trial.pt.

17. randomized controlled trial.pt.

18. multicenter study.pt.

19. clinical trial.pt.

20. exp Clinical Trials as topic/

21. or/6-20

22. (clinical adj trial$).tw.

23. ((singl$ or doubl$ or treb$ or tripl$) adj (blind$3 or mask$3)).tw.

24. PLACEBOS/

25. placebo$.tw.

26. randomly allocated.tw.

27. (allocated adj2 random$).tw.

28. or/22-27

29. 21 or 28

30. case report.tw.

31. letter/

32. historical article/

33. or/30-32

34. 29 not 33

35. 5 and 34
