# Supplementary material for: Randomised Trial Support for Orthopaedic Surgical Procedures
Source: PLoS One. 2014 Jun 13;9(6):e96745. doi: 10.1371/journal.pone.0096745 (PMC4057075; doi:10.1371/journal.pone.0096745)
Supplement: Appendix S3 — List of included and excluded RCTs after full-text assessment. (DOCX) [file pone.0096745.s003.docx]

**APPENDIX** **S3**

**List of included and excluded RCTs after full-text assessment**

**Knee Arthroscopy**

**Included:**

1. Arden, N. K., et al. (2008), 'A randomised controlled trial of tidal irrigation vs corticosteroid injection in knee osteoarthritis: the KIVIS Study', *Osteoarthritis Cartilage,* 16 (6), 733-9.
2. Chang, R. W., et al. (1993), 'A randomized, controlled trial of arthroscopic surgery versus closed-needle joint lavage for patients with osteoarthritis of the knee', *Arthritis Rheum,* 36 (3), 289-96.
3. Forster, M. C. and Straw, R. (2003), 'A prospective randomised trial comparing intra-articular Hyalgan injection and arthroscopic washout for knee osteoarthritis', *Knee,* 10 (3), 291-3.
4. Herrlin, S., et al. (2007), 'Arthroscopic or conservative treatment of degenerative medial meniscal tears: a prospective randomised trial', *Knee Surg Sports Traumatol Arthrosc,* 15 (4), 393-401.
5. Kalunian, K. C., et al. (2000), 'Visually-guided irrigation in patients with early knee osteoarthritis: a multicenter randomized, controlled trial', *Osteoarthritis Cartilage,* 8 (6), 412-8.
6. Kettunen, J. A., et al. (2007), 'Knee arthroscopy and exercise versus exercise only for chronic patellofemoral pain syndrome: a randomized controlled trial', *BMC Med,* 5, 38.
7. Kirkley, A., et al. (2008), 'A randomized trial of arthroscopic surgery for osteoarthritis of the knee', *N Engl J Med,* 359 (11), 1097-107.
8. Livesley, P. J., et al. (1991), 'Arthroscopic lavage of osteoarthritic knees', *J Bone Joint Surg Br,* 73 (6), 922-6.
9. Moseley, J. B., et al. (2002), 'A controlled trial of arthroscopic surgery for osteoarthritis of the knee', *N Engl J Med,* 347 (2), 81-8.
10. Xu, H. T., et al. (2008), '[Effect of various intervention factors on MMP-3 and TIMP-1 level in synovial fluid in knee joints with osteroarthritis]', *Zhong Nan Da Xue Xue Bao Yi Xue Ban,* 33 (1), 47-52.

**Excluded:**

1. Aydogan, N. H., et al. (2008), 'The effect of arthroscopic surgery and intraarticular drug injection to the antioxidation system and lipid peroxidation at osteoarthritis of knee', *Saudi Med J,* 29 (3), 397-402.

*Excluded as subjects in both intervention arms received arthroscopic surgery*

1. Moseley, J. B., Jr., et al. (1996), 'Arthroscopic treatment of osteoarthritis of the knee: a prospective, randomized, placebo-controlled trial. Results of a pilot study', *Am J Sports Med,* 24 (1), 28-34.

*Excluded as this was a pilot study*

**Internal Fixation of Proximal Fracture of the Femur**

**Included:**

1. Bong, S. C., et al. (1981), 'The treatment of unstable intertrochanteric fractures of the hip: a prospective trial of 150 cases', *Injury,* 13 (2), 139-46.
2. Hornby, R., Evans, J. G., and Vardon, V. (1989), 'Operative or conservative treatment for trochanteric fractures of the femur. A randomised epidemiological trial in elderly patients', *J Bone Joint Surg Br,* 71 (4), 619-23.

**Internal Fixation of Distal Radius Fracture**

**Included:**

1. Ekenstam, F., Jakobsson, O. P., and Wadin, K. (1989), 'Repair of the triangular ligament in Colles' fracture. No effect in a prospective randomized study', *Acta Orthop Scand,* 60 (4), 393-6.
2. Azzopardi, T., et al. (2005), 'Unstable extra-articular fractures of the distal radius: a prospective, randomised study of immobilisation in a cast versus supplementary percutaneous pinning', *J Bone Joint Surg Br,* 87 (6), 837-40.
3. Gupta, R., Raheja, A., and Modi, U. (1999), 'Colles' fracture: management by percutaneous crossed-pin fixation versus plaster of Paris cast immobilization', *Orthopedics,* 22 (7), 680-2.
4. Kapoor, H., Agarwal, A., and Dhaon, B. K. (2000), 'Displaced intra-articular fractures of distal radius: a comparative evaluation of results following closed reduction, external fixation and open reduction with internal fixation', *Injury,* 31 (2), 75-9.
5. McLauchlan, G. J., et al. (2002), 'Management of completely displaced metaphyseal fractures of the distal radius in children. A prospective, randomised controlled trial', *J Bone Joint Surg Br,* 84 (3), 413-7.
6. McQueen, M. M. (1998), 'Redisplaced unstable fractures of the distal radius. A randomised, prospective study of bridging versus non-bridging external fixation', *J Bone Joint Surg Br,* 80 (4), 665-9.
7. Rodriguez-Merchan, E. C. (1997), 'Plaster cast versus percutaneous pin fixation for comminuted fractures of the distal radius in patients between 46 and 65 years of age', *J Orthop Trauma,* 11 (3), 212-7.
8. Shankar, N. S. and Craxford, A. D. (1992), 'Comminuted Colles' fractures: a prospective trial of management', *J R Coll Surg Edinb,* 37 (3), 199-202.
9. Stoffelen, D. V. and Broos, P. L. (1998), 'Kapandji pinning or closed reduction for extra-articular distal radius fractures', *J Trauma,* 45 (4), 753-7.
10. Wong, T. C., et al. (2010), 'Casting versus percutaneous pinning for extra-articular fractures of the distal radius in an elderly Chinese population: a prospective randomised controlled trial', *J Hand Surg Eur Vol,* 35 (3), 202-8.
11. Zyluk, A. and Janowski, P. (2007), '[A comparison of the results of the conservative vs operative by percutaneous Kirschner-wiring treatment of fractures of the distal radius]', *Chir Narzadow Ruchu Ortop Pol,* 72 (5), 327-34.

**Excluded:**

1. Gibbons, C. L., et al. (1994), 'The management of isolated distal radius fractures in children', *J Pediatr Orthop,* 14 (2), 207-10.

*Excluded due to non-randomisation - treatment group determined according to which consultant was responsible for care.*

1. Kreder, H. J., et al. (2006), 'A randomized, controlled trial of distal radius fractures with metaphyseal displacement but without joint incongruity: closed reduction and casting versus closed reduction, spanning external fixation, and optional percutaneous K-wires', *J Orthop Trauma,* 20 (2), 115-21.

*Excluded as the operative intervention involved external fixation*

1. Stoffelen, D. V. and Broos, P. L. (1999), 'Closed reduction versus Kapandji-pinning for extra-articular distal radial fractures', *J Hand Surg Br,* 24 (1), 89-91.

*Exlcuded as the dataset was the same as that used in Stoffelen et al. 1998.*

**Ankle Fracture Fixation**

**Included:**

1. Bauer, M., et al. (1985), 'Malleolar fractures: nonoperative versus operative treatment. A controlled study', *Clin Orthop Relat Res,* (199), 17-27.
2. Buckley, R., et al. (2002), 'Operative compared with nonoperative treatment of displaced intra-articular calcaneal fractures: a prospective, randomized, controlled multicenter trial', *J Bone Joint Surg Am,* 84-A (10), 1733-44.
3. Makwana, N. K., et al. (2001), 'Conservative versus operative treatment for displaced ankle fractures in patients over 55 years of age. A prospective, randomised study', *J Bone Joint Surg Br,* 83 (4), 525-9.
4. Parmar, H. V., Triffitt, P. D., and Gregg, P. J. (1993), 'Intra-articular fractures of the calcaneum treated operatively or conservatively. A prospective study', *J Bone Joint Surg Br,* 75 (6), 932-7.
5. Phillips, W. A., et al. (1985), 'A prospective, randomized study of the management of severe ankle fractures', *J Bone Joint Surg Am,* 67 (1), 67-78.
6. Rowley, D. I., Norris, S. H., and Duckworth, T. (1986), 'A prospective trial comparing operative and manipulative treatment of ankle fractures', *J Bone Joint Surg Br,* 68 (4), 610-3.
7. Salai, M., et al. (2000), 'The epidemic of ankle fractures in the elderly--is surgical treatment warranted?', *Arch Orthop Trauma Surg,* 120 (9), 511-3.

**Excluded:**

1. Howard, J. L., et al. (2003), 'Complications following management of displaced intra-articular calcaneal fractures: a prospective randomized trial comparing open reduction internal fixation with nonoperative management', *J Orthop Trauma,* 17 (4), 241-9.

*Excluded as the dataset was the same as that used in Buckley et al. (2002).*

1. Ibrahim, T., et al. (2007), 'Displaced intra-articular calcaneal fractures: 15-year follow-up of a randomised controlled trial of conservative versus operative treatment', *Injury,* 38 (7), 848-55.

*Excluded as the dataset was the same as that used in Parmar et al. (1993).*

**Acromioplasty / repair of rotator cuff**

**Included:**

1. Brox, J. I., et al. (1993), 'Arthroscopic surgery compared with supervised exercises in patients with rotator cuff disease (stage II impingement syndrome)', *BMJ,* 307 (6909), 899-903.
2. Haahr, J. P., et al. (2005), 'Exercises versus arthroscopic decompression in patients with subacromial impingement: a randomised, controlled study in 90 cases with a one year follow up', *Ann Rheum Dis,* 64 (5), 760-4.
3. Ketola, S., et al. (2009), 'Does arthroscopic acromioplasty provide any additional value in the treatment of shoulder impingement syndrome?: a two-year randomised controlled trial', *J Bone Joint Surg Br,* 91 (10), 1326-34.
4. Moosmayer, S., et al. (2010), 'Comparison between surgery and physiotherapy in the treatment of small and medium-sized tears of the rotator cuff: A randomised controlled study of 103 patients with one-year follow-up', *J Bone Joint Surg Br,* 92 (1), 83-91.
5. Peters, Gabriela and Kohn, D. (1997), 'Medium-term clinical results after operative and non-operative treatment of subacromial impingement', *Der Unfallchirurg,* 100 (8), 623-29.

**Excluded:**

1. Brox, J. I., et al. (1999), 'Arthroscopic surgery versus supervised exercises in patients with rotator cuff disease (stage II impingement syndrome): a prospective, randomized, controlled study in 125 patients with a 2 1/2-year follow-up', *J Shoulder Elbow Surg,* 8 (2), 102-11.

*Excluded as the dataset was the same as that used in Brox et al. (1993).*

1. Haahr, J. P. and Andersen, J. H. (2006), 'Exercises may be as efficient as subacromial decompression in patients with subacromial stage II impingement: 4-8-years' follow-up in a prospective, randomized study', *Scand J Rheumatol,* 35 (3), 224-8.

*Excluded as the dataset was the same as that used in Haahr et al. (2005).*

1. Mohtadi, N. (2006), 'Exercises or arthroscopic decompression for subacromial impingement?', *Clin J Sport Med,* 16 (2), 193-4.

*Excluded as the dataset was the same as that used in Haahr et al. (2005).*

**Open Reduction of Fracture of Shaft of Tibia with Internal Fixation**

**Included:**

1. Abdel-Salam, A., Eyres, K. S., and Cleary, J. (1991), 'Internal fixation of closed tibial fractures for the management of sports injuries', *Br J Sports Med,* 25 (4), 213-7.
2. Hooper, G. J., Keddell, R. G., and Penny, I. D. (1991), 'Conservative management or closed nailing for tibial shaft fractures. A randomised prospective trial', *J Bone Joint Surg Br,* 73 (1), 83-5.
3. Karladani, A. H., et al. (2000), 'Displaced tibial shaft fractures: a prospective randomized study of closed intramedullary nailing versus cast treatment in 53 patients', *Acta Orthop Scand,* 71 (2), 160-7.
4. van der Linden, W. and Larsson, K. (1979), 'Plate fixation versus conservative treatment of tibial shaft fractures. A randomized trial', *J Bone Joint Surg Am,* 61 (6A), 873-8.

**Osteotomy**

**Included:**

1. Fulford, G. E., Lunn, P. G., and Macnicol, M. F. (1993), 'A prospective study of nonoperative and operative management for Perthes' disease', *J Pediatr Orthop,* 13 (3), 281-5.
2. Torkki, M., et al. (2001), 'Surgery vs orthosis vs watchful waiting for hallux valgus: a randomized controlled trial', *JAMA,* 285 (19), 2474-80.

**Open Reduction of Shoulder Dislocation**

**Included:**

1. Bottoni, C. R., et al. (2002), 'A prospective, randomized evaluation of arthroscopic stabilization versus nonoperative treatment in patients with acute, traumatic, first-time shoulder dislocations', *Am J Sports Med,* 30 (4), 576-80.
2. Edmonds, G., et al. (2003), 'The effect of early arthroscopic stabilization compared to nonsurgical treatment on proprioception after primary traumatic anterior dislocation of the shoulder', *Knee Surg Sports Traumatol Arthrosc,* 11 (2), 116-21.
3. Jakobsen, B. W., et al. (2007), 'Primary repair versus conservative treatment of first-time traumatic anterior dislocation of the shoulder: a randomized study with 10-year follow-up', *Arthroscopy,* 23 (2), 118-23.
4. Kirkley, A., et al. (1999), 'Prospective randomized clinical trial comparing the effectiveness of immediate arthroscopic stabilization versus immobilization and rehabilitation in first traumatic anterior dislocations of the shoulder', *Arthroscopy,* 15 (5), 507-14.
5. Wintzell, G., et al. (2000), 'Arthroscopic lavage speeds reduction in effusion in the glenohumeral joint after primary anterior shoulder dislocation: a controlled randomized ultrasound study', *Knee Surg Sports Traumatol Arthrosc,* 8 (1), 56-60.
6. Wintzell, G., et al. (1999b), 'Arthroscopic lavage reduced the recurrence rate following primary anterior shoulder dislocation. A randomised multicentre study with 1-year follow-up', *Knee Surg Sports Traumatol Arthrosc,* 7 (3), 192-6.

**Excluded:**

1. Kirkley, A., et al. (2005), 'Prospective randomized clinical trial comparing the effectiveness of immediate arthroscopic stabilization versus immobilization and rehabilitation in first traumatic anterior dislocations of the shoulder: long-term evaluation', *Arthroscopy,* 21 (1), 55-63.

*Excluded as the dataset was the same as that used in Kirkley et al. (1999).*

1. Wintzell, G., et al. (1999a), 'Arthroscopic lavage compared with nonoperative treatment for traumatic primary anterior shoulder dislocation: a 2-year follow-up of a prospective randomized study', *J Shoulder Elbow Surg,* 8 (5), 399-402.

*Excluded as the dataset was a subset of that used in Wintzell et al. (1999b).*

1. Wintzell, G., et al. (1996), 'A prospective controlled randomized study of arthroscopic lavage in acute primary anterior dislocation of the shoulder: one-year follow-up', *Knee Surg Sports Traumatol Arthrosc,* 4 (1), 43-7.

*Excluded as the dataset was a subset of that used in Wintzell et al. (1999b).*

**Open Reduction of Acromioclavicular Dislocation**

**Included:**

1. Bannister, G. C., et al. (1989), 'The management of acute acromioclavicular dislocation. A randomised prospective controlled trial', *J Bone Joint Surg Br,* 71 (5), 848-50.
2. Imatani, R. J., Hanlon, J. J., and Cady, G. W. (1975), 'Acute, complete acromioclavicular separation', *J Bone Joint Surg Am,* 57 (3), 328-32.
3. Larsen, E., Bjerg-Nielsen, A., and Christensen, P. (1986), 'Conservative or surgical treatment of acromioclavicular dislocation. A prospective, controlled, randomized study', *J Bone Joint Surg Am,* 68 (4), 552-5.

**Open Reduction of Patella Dislocation**

**Included:**

1. Camanho, G. L., et al. (2009), 'Conservative versus surgical treatment for repair of the medial patellofemoral ligament in acute dislocations of the patella', *Arthroscopy,* 25 (6), 620-5.
2. Christiansen, S. E., et al. (2008), 'Isolated repair of the medial patellofemoral ligament in primary dislocation of the patella: a prospective randomized study', *Arthroscopy,* 24 (8), 881-7.
3. Nikku, R., et al. (1997), 'Operative versus closed treatment of primary dislocation of the patella. Similar 2-year results in 125 randomized patients', *Acta Orthop Scand,* 68 (5), 419-23.
4. Palmu, S., et al. (2008), 'Acute patellar dislocation in children and adolescents: a randomized clinical trial', *J Bone Joint Surg Am,* 90 (3), 463-70.

**Excluded:**

1. Nietosvaara, Y., et al. (2009), 'Acute patellar dislocation in children and adolescents. Surgical technique', *J Bone Joint Surg Am,* 91 Suppl 2 Pt 1, 139-45.

*Excluded as the dataset was the same as that used in Palmu et al. (2008).*

1. Nikku, R., et al. (2005), 'Operative treatment of primary patellar dislocation does not improve medium-term outcome: A 7-year follow-up report and risk analysis of 127 randomized patients', *Acta Orthop,* 76 (5), 699-704.

*Excluded as the dataset was the same as that used in Nikku et al. (1997).*

**Knee, Repair of Cruciate Ligament**

**Included:**

1. Andersson, C. and Gillquist, J. (1992), 'Treatment of acute isolated and combined ruptures of the anterior cruciate ligament. A long-term follow-up study', *Am J Sports Med,* 20 (1), 7-12.
2. Odensten, M., et al. (1985), 'Surgical or conservative treatment of the acutely torn anterior cruciate ligament. A randomized study with short-term follow-up observations', *Clin Orthop Relat Res,* (198), 87-93.
3. Sandberg, R., et al. (1987), 'Operative versus non-operative treatment of recent injuries to the ligaments of the knee. A prospective randomized study', *J Bone Joint Surg Am,* 69 (8), 1120-6.

**Excluded:**

1. Andersson, C., et al. (1989), 'Surgical or non-surgical treatment of acute rupture of the anterior cruciate ligament. A randomized study with long-term follow-up', *J Bone Joint Surg Am,* 71 (7), 965-74.

*Excluded as the dataset was a subset of that used in Andersson et al. (1992).*

1. Andersson, C., Odensten, M., and Gillquist, J. (1991), 'Knee function after surgical or nonsurgical treatment of acute rupture of the anterior cruciate ligament: a randomized study with a long-term follow-up period', *Clin Orthop Relat Res,* (264), 255-63.
2. Meunier, A., Odensten, M., and Good, L. (2007), 'Long-term results after primary repair or non-surgical treatment of anterior cruciate ligament rupture: a randomized study with a 15-year follow-up', *Scand J Med Sci Sports,* 17 (3), 230-7.

*Excluded as the dataset was the same as that used in Odensten et al. (1985).*

**Repair of Achilles Tendon Rupture**

**Included:**

1. Cetti, R., et al. (1993), 'Operative versus nonoperative treatment of Achilles tendon rupture. A prospective randomized study and review of the literature', *Am J Sports Med,* 21 (6), 791-9.
2. Majewski, M., Rickert, M., and Steinbruck, K. (2000), '[Achilles tendon rupture. A prospective study assessing various treatment possibilities]', *Orthopade,* 29 (7), 670-6.
3. Metz, R., et al. (2008), 'Acute Achilles tendon rupture: minimally invasive surgery versus nonoperative treatment with immediate full weightbearing--a randomized controlled trial', *Am J Sports Med,* 36 (9), 1688-94.
4. Moller, M., et al. (2001), 'Acute rupture of tendon Achillis. A prospective randomised study of comparison between surgical and non-surgical treatment', *J Bone Joint Surg Br,* 83 (6), 843-8.
5. Nilsson-Helander, K., et al. (2010), 'Acute achilles tendon rupture: a randomized, controlled study comparing surgical and nonsurgical treatments using validated outcome measures', *Am J Sports Med,* 38 (11), 2186-93.
6. Nistor, L. (1981), 'Surgical and non-surgical treatment of Achilles Tendon rupture. A prospective randomized study', *J Bone Joint Surg Am,* 63 (3), 394-9.
7. Thermann, H., Zwipp, H., and Tscherne, H. (1995), '[Functional treatment concept of acute rupture of the Achilles tendon. 2 years results of a prospective randomized study]', *Der Unfallchirurg,* 98 (1), 21-32.
8. Twaddle, B. C. and Poon, P. (2007), 'Early motion for Achilles tendon ruptures: is surgery important? A randomized, prospective study', *Am J Sports Med,* 35 (12), 2033-8.
9. Willits, K., et al. (2010), 'Operative versus nonoperative treatment of acute Achilles tendon ruptures: a multicenter randomized trial using accelerated functional rehabilitation', *J Bone Joint Surg Am,* 92 (17), 2767-75.

**Excluded:**

1. Costa, M. L., et al. (2006), 'Randomised controlled trials of immediate weight-bearing mobilisation for rupture of the tendo Achillis', *J Bone Joint Surg Br,* 88 (1), 69-77.
2. Metz, R., et al. (2009), 'Recovery of calf muscle strength following acute achilles tendon rupture treatment: a comparison between minimally invasive surgery and conservative treatment', *Foot Ankle Spec,* 2 (5), 219-26.

*Excluded as the dataset was the same as that used in Metz et al. (2008).*

1. Moller, M., et al. (2002a), 'Calf muscle function after Achilles tendon rupture. A prospective, randomised study comparing surgical and non-surgical treatment', *Scand J Med Sci Sports,* 12 (1), 9-16.

*Excluded as the dataset was the same as that used in Moller et al. (2001).*

1. Moller, M., et al. (2002b), 'The ultrasonographic appearance of the ruptured Achilles tendon during healing: a longitudinal evaluation of surgical and nonsurgical treatment, with comparisons to MRI appearance', *Knee Surg Sports Traumatol Arthrosc,* 10 (1), 49-56.

*Excluded as the dataset was a subset of that used in Moller et al. (2001).*

**Humerus, Distal, Treatment of Fracture by Open Reduction with Internal Fixation**

**Included:**

1. Pandey, S., et al. (2008), 'Treatment of supracondylar fracture of the humerus (type IIB and III) in children: A prospective randomized controlled trial comparing two methods', *Kathmandu Univ Med J (KUMJ),* 6 (23), 310-8.

**Excluded:**

1. Nast-Kolb, D., Knoefel, W. T., and Schweiberer, L. (1991), '[The treatment of humeral shaft fractures. Results of a prospective AO multicenter study]', *Der Unfallchirurg,* 94 (9), 447-54.

*Excluded due to non-randomisation. The process of randomisation and treatment arm allocation was not stated.*

1. Wallny, T., et al. (1997), 'Comparative results of bracing and interlocking nailing in the treatment of humeral shaft fractures', *Int Orthop,* 21 (6), 374-9.

*Excluded on the basis of study desgin. The study as a retrospective case-control study.*

**Joint arthrodesis**

**Included:**

1. Wood, K., et al. (2003), 'Operative compared with nonoperative treatment of a thoracolumbar burst fracture without neurological deficit. A prospective, randomized study', *J Bone Joint Surg Am,* 85-A (5), 773-81.

**Abscess Drainage**

**Included:**

1. Ernst, A. A., Marvez-Valls, E., and Martin, D. H. (1995), 'Incision and drainage versus aspiration of fluctuant buboes in the emergency department during an epidemic of chancroid', *Sex Transm Dis,* 22 (4), 217-20.
2. Eryilmaz, R., et al. (2005), 'Management of lactational breast abscesses', *Breast,* 14 (5), 375-9.
3. Singh, J. P. and Kashyap, A. (1989), 'A comparative evaluation of percutaneous catheter drainage for resistant amebic liver abscesses', *Am J Surg,* 158 (1), 58-62.
4. Spires, J. R., et al. (1987), 'Treatment of peritonsillar abscess. A prospective study of aspiration vs incision and drainage', *Arch Otolaryngol Head Neck Surg,* 113 (9), 984-6.
5. Stringer, S. P., Schaefer, S. D., and Close, L. G. (1988), 'A randomized trial for outpatient management of peritonsillar abscess', *Arch Otolaryngol Head Neck Surg,* 114 (3), 296-8.

**Clavicle, Treatment of Fracture, Open Reduction with Internal Fixation**

**Included:**

1. Altamimi, S. A. and McKee, M. D. (2008), 'Nonoperative treatment compared with plate fixation of displaced midshaft clavicular fractures. Surgical technique', *J Bone Joint Surg Am,* 90 Suppl 2 Pt 1, 1-8.
2. Judd, D. B., et al. (2009), 'Acute operative stabilization versus nonoperative management of clavicle fractures', *Am J Orthop (Belle Mead NJ),* 38 (7), 341-5.
3. Smekal, V., et al. (2009), 'Elastic stable intramedullary nailing versus nonoperative treatment of displaced midshaft clavicular fractures-a randomized, controlled, clinical trial', *J Orthop Trauma,* 23 (2), 106-12.

**Excluded:**

1. Jubel, A., et al. (2005), 'Die Behandlung der diaphysären Klavikulafraktur', *Der Unfallchirurg,* 108 (9), 707-14.

*Excluded due to non-randomisation. The process of randomisation and treatment arm allocation was not stated.*

**Humerus, Proximal, Treatment of Fracture, Open Reduction with Internal Fixation**

**Included:**

1. Fjalestad, T., et al. (2010), 'Health and cost consequences of surgical versus conservative treatment for a comminuted proximal humeral fracture in elderly patients', *Injury,* 41 (6), 599-605.
2. Zyto, K., et al. (1997), 'Treatment of displaced proximal humeral fractures in elderly patients', *J Bone Joint Surg Br,* 79 (3), 412-7.

**Excluded:**

1. Brorson, S., et al. (2009), 'Effect of osteosynthesis, primary hemiarthroplasty, and non-surgical management for displaced four-part fractures of the proximal humerus in elderly: a multi-centre, randomised clinical trial', *Trials,* 10, 51.

*Excluded as this was a study protocol only.*

1. Den Hartog, D., et al. (2010), 'Primary hemiarthroplasty versus conservative treatment for comminuted fractures of the proximal humerus in the elderly (ProCon): a multicenter randomized controlled trial', *BMC Musculoskelet Disord,* 11, 97.

*Excluded as this was a study protocol only.*

**Release of Carpal Tunnel**

**Included:**

1. Garland, H., et al. (1964), 'SURGICAL TREATMENT FOR THE CARPAL TUNNEL SYNDROME', *Lancet,* 1 (7343), 1129-30.
2. Gerritsen, A. A., et al. (2002), 'Splinting vs surgery in the treatment of carpal tunnel syndrome: a randomized controlled trial', *JAMA,* 288 (10), 1245-51.
3. Jarvik, J. G., et al. (2009), 'Surgery versus non-surgical therapy for carpal tunnel syndrome: a randomised parallel-group trial', *Lancet,* 374 (9695), 1074-81.
4. Korthals-de Bos, I. B., et al. (2006), 'Surgery is more cost-effective than splinting for carpal tunnel syndrome in the Netherlands: results of an economic evaluation alongside a randomized controlled trial', *BMC Musculoskelet Disord,* 7, 86.
5. Ly-Pen, D., et al. (2005), 'Surgical decompression versus local steroid injection in carpal tunnel syndrome: a one-year, prospective, randomized, open, controlled clinical trial', *Arthritis Rheum,* 52 (2), 612-9.

**Excluded:**

1. Martin, B. I., et al. (2005), 'Randomized clinical trial of surgery versus conservative therapy for carpal tunnel syndrome [ISRCTN84286481]', *BMC Musculoskelet Disord,* 6, 2.

*Excluded as this was a study protocol only.*
