# Supplementary material for: Randomised Trial Support for Orthopaedic Surgical Procedures
Source: PLoS One. 2014 Jun 13;9(6):e96745. doi: 10.1371/journal.pone.0096745 (PMC4057075; doi:10.1371/journal.pone.0096745)
Supplement: Appendix S4 — Characteristics of included RCTs. (DOCX) [file pone.0096745.s004.docx]

**APPENDIX** **S4**

**Characteristics of included RCTs**

| **Procedure Category and Study** | **Sample size & Population** | | **Specific Comparison** | | | **Power Calculation Performed** (Y/N) | **Primary Outcome Specified**  (Y/N) | **Result of Primary Outcome** (Supportive/Not Supportive/Equivocal) | | **Other Outcomes Measured (Categorised) & Results Of Measured Outcomes** (Significantly Supportive of Surgery - Y/N)* |
| --- | --- | --- | --- | --- | --- | --- | --- | --- | --- | --- |
|  | | | | | | | | | | |
| **Knee Arthroscopy** | | | | | | | | | | |
| 1. Arden 2008 | 150 adults with knee OA | | Tidal irrigation vs. corticoid steroid injection | | | Yes | Yes – WOMAC pain score | Supportive of operative procedure | | 1. Functional – Y 2. Self-reported improvement – Y 3. 50m walk and stair-climb time - N |
|  | Conclusion: Both procedures lead to significant short-term pain relief of at least 4 weeks, however, TI displayed a significantly greater duration of benefit. | | | | | | | | | |
| 2. Chang 1993 | 32 adults with knee OA refractory to conservative management | | Arthroscopic surgery vs. closed needle joint lavage | | | No | No | NA | | 1. Clinical parameters – N 2. Self-reported pain and functional status - N 3. Walk time - N 4. Global scales - N 5. Medical costs - N |
|  | Conclusion: The search for and removal of soft tissue abnormalities via arthroscopic surgery does not appear justified for all patients with non-end-stage OA of the knee who fail to respond to conservative therapy, but it may be beneficial for certain subgroups. | | | | | | | | | |
| 3. Forster 2003 | 38 adults with knee OA | | Arthroscopic washout vs. intra-articular Hyalgan injections | | | No | No | NA | | 1. Pain – N 2. Functional - N |
|  | Conclusion: The use of intra-articular Hyalgan injections in patients with knee osteoarthritis without mechanical symptoms gives results comparable with arthroscopic washout. | | | | | | | | | |
| 4. Herrlin 2006 | 90 adults with atraumatic knee pain | | Arthroscopic partial meniscectomy + supervised exercise or supervised exercise alone | | | Yes | Yes – Knee Injury and Osteoarthritis Outcome Score (KOOS) | | Not supportive of operative procedure | 1. Global- N 2. Functional – N 3. Pain - N |
|  | Conclusion: Arthroscopic partial medial meniscectomy followed by supervised exercise was not superior to supervised exercise alone in terms of reduced knee pain, improved knee function and improved quality of life. | | | | | | | | | |
| 5. Kalunian 2000 | 90 adults with early knee OA | | Arthroscopic irrigation with 3000 ml of saline (treatment group) vs. minimal amount of irrigation (250 ml) required to perform arthroscopy (placebo group) | | | Yes | Yes – change in aggregate WOMAC pain score | | Not supportive of operative procedure | 1. Pain (WOMAC) – Y 2. Pain (VAS) - Y |
|  | Conclusion: Visually-guided arthroscopic irrigation may be a useful therapeutic option for relief of pain in a subset of patients with knee OA, particularly in those who have occult intra-articular crystals. | | | | | | | | | |
| 6. Kettunen 2007 | 56 adults with chronic patellofemoral pain syndrome | | | Knee arthroscopy and 8-week home exercise program vs. 8-week home exercise program only | | Yes | Yes - Kujala score (Anterior Knee Score) | | Not supportive of operative procedure | 1. Pain – N 2. Global - N |
|  | Conclusion: The outcome when arthroscopy was used in addition to a home exercise program was no better than when the home exercise program was used alone. | | | | | | | | | |
| 7. Kirkley 2008 | 188 adults with moderate to severe knee OA | | | Surgical lavage and arthroscopic debridement together with physical and medical therapy vs. treatment with physical and medical therapy alone | | Yes | Yes – WOMAC score | | Not supportive of operative procedure | 1. Quality of life – N 2. Functional - N |
|  | Conclusion: Arthroscopic surgery for osteoarthritis of the knee provides no additional benefit to optimized physical and medical therapy. | | | | | | | | | |
| 8. Livesley 1991 | 61 adults with knee OA | | | Arthroscopic lavage and physiotherapy vs. physiotherapy alone | | No | No | | NA | 1. Pain on activity – Y 2. Pain at night – Y 3. Pain on rest – N 4. Clinical – N 5. Self-assessment of improvement – N |
|  | Conclusion: Our results confirm the effectiveness of joint lavage in the management of painful osteoarthritis of the knee. | | | | | | | | | |
| 9. Moseley 2002 | 180 adults with knee OA | | | Arthroscopic debridement vs. arthroscopic lavage vs. placebo surgery | | Yes | Yes - Knee - Specific Pain Scale (KSPS) | | Not supportive of operative procedure | 1. Arthritis pain in general (AIMS2-P) - N  2. Body pain - N  3. Function - N |
|  | Conclusion: Outcomes after arthroscopic lavage or arthroscopic debridement were no better than those after a placebo procedure. | | | | | | | | | |
| 10. Xu 2008 | 60 patients with OA of the knee | | | Intra-articular hyaluronic acid vs. arthroscopic debridement vs. hyaluronic acid + glucosamine sulphate | | No | No | | NA | 1. MMP-3 levels – N 2. TIMP-1 – N 3. MMP/TIMP-1 ratio – N |
|  | Conclusion: (1) HA, GS and AD all can decrease the level of MMP-3 and the ratio of MMP-3/TIMP-1 in the synovial fluid in knee joints with OA. (2) The level of TIMP-1 in the synovial fluid has no difference before and after being treated with HA. The AD group and GS group can increase the level of TIMP-1, which indicates that the AD group or GS group might produce better therapeutic effect. (3) The level of TIMP-1 increased in the AD group is more than that in the HA' group and the GS+HA' group after being treated for 4 weeks, which indicates that the AD group might get better therapeutic effect than the other 2 groups. | | | | | | | | | |
| **Internal Fixation of Proximal Fracture of the Femur** | | | | | | | | | | |
| 1. Bong 1981 | 150 adults with unstable intertrochanteric fractures of the proximal femur | | | Skeletal traction with a tibial pin vs. medial displacement osteotomy vs. valgus osteotomy- | | No | No | | NA | 1. Functional – N 2. Anatomical – N 3. Complications – N |
|  | Conclusion: 1. Conservative management of unstable intertrochanteric fractures is well tolerated by Chinese patients but requires a good standard of nursing care. 2. There is no significant difference between the functional results in patients treated with the Dimon and Hughston osteotomy and those treated by Sarmiento osteotomy. 3. An infection rate of 4 per cent was found in the two operative groups of patients. 4. The McLaughlin pin and plate is not an ideal fixation apparatus for unstable intertrochanteric fractures. 5. One case of avascular necrosis is reported. | | | | | | | | | |
| 2. Hornby 1989 | 106 adults with extra-capsular fractures of the hip | | | Internal fixation with the AO dynamic hip screw vs. Hamilton tibial pin traction | | No | No | | NA | 1. Overall outcome (Discharged/deceased/in hospital) – N 2. Length of Stay – Y 3. Complications – N 4. Anatomical - Y |
|  | Conclusion: Operative treatment gave better anatomical results and a shorter hospital stay, but significantly more of the patients treated by traction showed loss of independence six months after injury. | | | | | | | | | |
| **Internal Fixation of Distal Radius Fracture** | | | | | | | | | | |
| 1. Azzopardi 2005 | | 57 adults with unstable extra-articular fractures of the distal radius. | | Immobilisation in a cast alone vs. immobilization with cast and supplementary percutaneous pinning | | No | No | | NA | 1. Clinical – N 2. Radiological – N 3. Functional – N |
|  |  | Conclusion: Percutaneous pinning of unstable, extra-articular fractures of the distal radius provides only a marginal improvement in the radiological parameters compared with immobilisation in a cast alone. This does not correlate with an improved functional outcome in a low-demand, elderly population. | | | | | | | | |
| 2. Ekenstam 1989 | | 41 patients with extra-articular Colles’ fracture with a fractured ulnar styloid. | | Closed manipulation and an above-the-elbow plaster cast vs. closed reduction + transfixation of avulsed ulnar styloid +/- repair of triangular ligament | | No | No | | NA | 1. Radiological - Y 2. Clinical - N |
|  |  | Conclusion: Repair of the ruptured triangular ligament in extra-articular fractures of the distal radius is not better than conventional treatment. | | | | | | | | |
| 3. Gupta 1999 | | 50 patient (22-80 years of age) with Colles’ fracture after fusion of epiphysis | | Percutaneous crossed-pin fixation + plaster of Paris cast immobilisation s. conventional plaster of Paris cast immobilisation | | No | No | | NA | 1. Anatomical – N 2. Functional - N |
|  |  | Conclusion: The anatomical and functional end results were significantly better with percutaneous crossed-pin fixation at final follow up. | | | | | | | | |
| 4. Kapoor 2000 | | 90 adults with acute displaced intra-articular fractures of the lower end of the radius. | | Closed reduction and plaster immobilization vs. external fixation vs. open reduction and internal fixation | | No | No | | NA | 1. Anatomical – N 2. Clinical – Y 3. Functional – N 4. Complications – N |
|  |  | Conclusion: Primary operative treatment generates significantly better anatomical and functional results than closed reduction and casting. | | | | | | | | |
| 5. McLauchlan 2002 | | 68 children with completely displaced metaphyseal fractures of the distal radius | | Manipulation (MUA) and application of an above-elbow cast alone vs. MUA + insertion of a percutaneous Kirschner (K-) wire | | No | No | | NA | 1. Radiological – Y 2. Clinical – N 3. Need for second procedure - Y |
|  |  | Conclusion: Supplementary percutaneous K-wire fixation resulted in a significantly better maintenance of the alignment of the fracture. It was safe and reduced the need for follow-up radiographs and further procedures to correct loss of position with no detrimental effect on the outcome. | | | | | | | | |
| 6. McQueen 1996 | | 120 adults with redisplaced fractures of the distal radius | | Manipulation and plaster vs. open reduction and bone grafting vs. closed external fixation +/- mobilisation of the wrist at three weeks | | No | No | | NA | 1. Anatomical – Y 2. Functional – N |
|  |  | Conclusion: The radiological results showed improvement in angulation of the distal radius for the open reduction and bone grafting group. Functional results at six weeks, three and six months and at one year, however, showed no difference between any of the four groups. | | | | | | | | |
| 7. Rodríguez-Merchán 1997 | | 40 adults with unstable distal radius fractures | | | Closed reduction + cast immobilisation vs. percutaneous K-wires and cast | No | No | | NA | 1. Anatomical – N 2. Functional – N |
|  |  | Conclusion: Functional results in the pinning group were better (excellent, 12; good, 6; fair, 2) than in the plaster group (excellent, 3; good, 8; fair, 5; poor, 4). Anatomic results also were better in the pinning group. | | | | | | | | |
| 8. Shankar 1992 | | 45 adults with comminuted distal radius fractures | | Percutaneous wiring vs. manipulation and application of plaster of Paris | | No | Yes – McBride score | | Supportive of operative procedure | 1. Functional – Y 2. Radiological – Y 3. Complications - N |
|  |  | Conclusion: There were statistically significant differences between the two groups at 6 months in extension (P less than 0.001), ulnar deviation (P greater than 0.05), grip strength (P less than 0.001), radial angle (P less than 0.0001), radial length (P less than 0.0001), and dorsal/volar angle (P greater than 0.0001). Using a modified McBride's system of scoring, the results were found to be directly correlated to the anatomical findings at the end of 6 months. | | | | | | | | |
| 9. Stoffelen 1998 | | 98 adults with extra-articular distal radial fractures | | Closed reduction vs. kapandji-pinning | | No | No | | NA | 1. Functional – N 2. Clinical - N |
|  |  | Conclusion: In terms of maintenance of reduction and functional outcome at 1-year follow-up, no statistically significant differences were found between the two groups. We conclude, therefore, that both techniques can be applied to extra-articular fractures of the distal radius according to the characteristics of the forearm and the surgeon's or the patient's need. | | | | | | | | |
| 10. Wong 2010 | | 60 adults with extra-articular distal radius fractures | | Casting vs. percutaneous pinning | | No | No | | NA | 1. Functional – N 2. QOL - N 3. Radiological – Y 4. Complications - N |
|  |  | Conclusion: The radiological outcomes in terms of dorsal angulation, radial inclination and radial length were statistically significantly better in the K-wire group, whereas the Mayo wrist score and quality of life, healing rate, healing time, and complications were similar. The functional outcomes and quality of life were not affected by the treatments. Both treatments had a very low rate of complication and high healing rates. | | | | | | | | |
| 11. Zyluk 2007 | | 60 adults with isolated displaced fractures of the distal radius | | Conservative vs. Kirschner wiring | | Not stated in abstract | Yes - rate of secondary displacement | | Supportive of operative procedure | 1. Functional – Y 2. Radiological – N |
|  |  | Conclusion: Percutaneous K-wiring of fractures of the distal radius Is superior to the conservative treatment because statistically significantly reduces the risk of secondary displacement and allows to obtain stronger grip and better hand function within 6 months after fracture. | | | | | | | | |
| **Ankle Fracture Fixation** | | | | | | | | | | |
| 1. Bauer 1985 | 98 skeletally mature patients with intra-articular malleolar fractures | | | ORIF vs. closed reduction and plaster cast | | No | No | | NA | 1. Radiological - Y 2. Clinical - N 3. Functional - N   Time to return to work - N |
|  | Conclusion: The initial course was more favorable in surgically reduced fractures. However, follow- up examinations showed little difference in results between the two forms of treatment. | | | | | | | | | |
| 2. Buckley 2002 | 424 patients (15-68 years of age) with displaced intra-articular calcaneal fractures | | | Open reduction and internal fixation vs. non-operative management (ice, elevation + rest) | | Yes | Yes   1. SF-36 2. VAS | | Not supportive of operative procedure | 1. Radiological - N |
|  | Conclusion: Without stratification of the groups, the functional results after nonoperative care of displaced intra- articular calcaneal fractures were equivalent to those after operative care. However, after unmasking the data by removal of the patients who were receiving Workers’ Compensation, the outcomes were significantly better in some groups of surgically treated patients. | | | | | | | | | |
| 3. Makwana 2001 | 47 patients over 55 years of age with displaced ankle fractures | | | Open reduction and internal fixation vs. closed reduction + cast | | No | No | | NA | 1. Radiological – Y 2. Functional/Clinical – Y |
|  | Conclusion: We recommend treating displaced ankle fractures in patients over the age of 55 years by open reduction and internal fixation. | | | | | | | | | |
| 4. Parmar 1993 | 66 patients with intra-articular calcaneal fractures | | | Open reduction and internal fixation vs. conservative treatment (ice and elevation) | | No | No | | NA | 1. Pain – N 2. Functional – N |
|  | Conclusion: There was no significant difference in outcome between the operatively and the conservatively treated displaced fractures. | | | | | | | | | |
| 5. Phillips 1985 | 138 patients with grade-4 Lauge-Hansen ankle fractures | | | Open reduction and internal fixation vs. closed reduction + cast | | No | Yes - Authors’ 150-point scoring system | | Supportive of operative procedure | (Sub-components of scoring system)   1. Clinical score – N 2. Anatomical score – Y 3. Arthritis score – N 4. Total score - Y |
|  | Conclusion: Statistical analysis of the  data showed that, of the patients with initial satisfactory closed reduction, the ones treated by open reduction and  rigid internal fixation had significantly higher total  scores. | | | | | | | | | |
| 6. Rowley 1986 | 42 patients (16-70 years of age) with displaced ankle fractures | | | Open reduction and internal fixation vs. Non-manipulative reduction and plaster | | No | No | | NA | 1. Functional – N |
|  | Conclusion: There appears to be no difference in the outcome of treatment of the two groups in the early recovery period. | | | | | | | | | |
| 7. Salai 2000 | 84 patients over the age of 65 years with displaced ankle fractures | | | Open reduction and internal fixation vs. closed manipulation and cast | | No | Yes – AOFAS Ankle score | | Not supportive of operative procedure | 1. Costs of treatment – N |
|  | Conclusion: Conservative rather than operative treatment can result in a better functional outcome and save expensive, frequent surgical procedures. Furthermore, the cost of treatment per patient is far less in the non-operative group. | | | | | | | | | |
| **Acromioplasty / Repair of Rotator Cuff** | | | | | | | | | | |
| 1. Brox 1993 | 125 patients (18-66 years) with rotator cuff disease refractory to conservative treatment | | | Arthroscopic subacromial decompression vs. supervised exercise regimen vs. detuned soft laser treatment | | No | Yes – Neer Shoulder Score | | Not supportive of operative procedure | 1. Pain – N |
|  | Conclusion: Surgery or a supervised exercise regimen significantly, and equally, improved rotator cuff disease compared with placebo. | | | | | | | | | |
| 2. Haahr 2005 | 90 patients (18-55 years) with rotator cuff disease | | | Arthroscopic subacromial decompression vs. physiotherapeutic treatment | | Yes | Yes – Constant Score | | Not supportive of operative procedure | 1. Self-reported symptoms (Likert Scale) – N |
|  | Conclusion: Surgical treatment of rotator cuff syndrome with subacromial impingement was not superior to physiotherapy with training. | | | | | | | | | |
| 3. Ketola 2009 | 140 patients with shoulder impingement symptoms refractory to conservative treatment | | | Supervised exercise vs. arthroscopic acromioplasty + exercise | | Yes | Yes – Pain (VAS score) | | Not supportive of operative procedure | 1. Subcomponents of VAS – N 2. Shoulder Disability Questionnaire – N 3. Cost-effectiveness – N |
|  | Conclusion: Arthroscopic acromioplasty provides no clinically important effects over a structured and supervised exercise programme alone in terms of subjective outcome or cost-effectiveness when measured at 24 months. Structured exercise treatment should be the basis for treatment of shoulder impingement syndrome, with operative treatment offered judiciously until its true merit is proven. | | | | | | | | | |
| 4. Moosmayer 2010 | 103 patients with symptomatic small and medium-sized tears of the rotator cuff | | | Operative repair vs. physiotherapy | | Yes | Yes – Constant Score | | Supportive of operative procedure | 1. American Shoulder and Elbow Surgeons score - Y  2. SF-36 score – N  3. Patient Satisfaction – Y |
|  | Conclusion: Analysis of between-group differences showed better results for the surgery group on the Constant scale (difference 13.0 points, p - 0.002), on the American Shoulder and Elbow surgeons scale (difference 16.1 points, p < 0.0005), for pain-free abduction (difference 28.8°, p = 0.003) and for reduction in pain (difference on a visual analogue scale -1.7 cm, p < 0.0005). | | | | | | | | | |
| 5. Peters 1997 | 72 patients suffering from grade II subacromial impingement syndrome | | | Arthroscopic subacromial decompression vs. non-operative treatment | | No | Subjective Shoulder Rating Scare | | Not supportive of operative procedure | No |
|  | Conclusion: In summary, both forms of treatment led to an improvement of the subacromial impingement. The improvement in pain was the most marked. The long-term results are required for the final evaluation. | | | | | | | | | |
| **Open Reduction of Fracture of Shaft of Tibia with Internal Fixation** | | | | | | | | | | |
| 1. Abdel-Salam 1991 | 90 adults with closed tibial fractures | | | Open reduction and internal fixation vs. manipulation and cast | | No | No | | NA | 1. Radiological – Y 2. Return to normal activity – Y 3. Complications - N |
|  | Conclusion: It is concluded that internal fixation of closed tibial fractures as a primary procedure following low velocity sports injuries can be safely performed. It leads to a faster return to normal activities with fewer complications than conservative treatment in plaster using contemporary methods. | | | | | | | | | |
| 2. Hooper 1991 | 62 skeletally mature patients with displaced fracture of the tibial shaft | | | IM nail vs. closed manipulation + cast | | No | No | | NA | 1. Radiological – Y 2. Functional – N 3. Time in hospital – N 4. Time off work - Y   Complications – equivocal? |
|  | Conclusion: In our opinion, displaced fractures of the tibial shaft area better and more efficiently treated by closed intra-medullary nailing; this method has an acceptable complication rate when it is compared with conservative treatment. | | | | | | | | | |
| 3. Karladani 2000 | 53 adults with displaced and closed tibial shaft fractures | | | IM nailing vs. closed reduction + cast | | No | No | | NA | 1. NHP score – Y 2. Pain (VAS) – N   Complications - N |
|  | Conclusion: The Nottingham Health Profile index scores on physical mobility, social isolation, work ability, and sexual life were significantly better in group I than in group II at 3 months after injury. Delayed union, malunion, and restricted range of motion at the ankle joint were common complications when these fractures were treated with a cast. We recommend intramedullary nailing for these fractures. | | | | | | | | | |
| 4. Van Der Linden 1979 | 100 adults with displaced fractures of the tibial shaft (open and closed) | | | AO –plate vs. closed reduction + cast | | No | No | | NA | 1. Complications – N 2. Time in hospital – N 3. Time to healing – Y 4. Anatomical – Y   Functional – Y |
|  | Conclusion: Summarizing our findings, both methods were found to have advantages and drawbacks. There was a conclusive advantage in conservative treatment for open fractures and in AO-plate treatment for closed longitudinal fractures. | | | | | | | | | |
| **Osteotomy** | | | | | | | | | | |
| 1. Fulford 1993 | 94 paediatric patients with symptomatic Perthes disease | | | Bed rest and skin traction + weight relieving calliper vs. proximal femoral varus osteotomy | | No | No | | NA | 1. Shape of femoral head (Catterall grouping) - N 2. Clinical - N 3. Functional - N |
|  | Conclusion: The outcome was similar in both groups and could be predicted more effectively by the arthrographic shape of the femoral head at presentation than by the Catterall grouping. | | | | | | | | | |
| 2. Torkki 2001 | 209 patients with painful bunion and a hallux valgus angle 35 degrees or less | | | Surgery (distal chevron osteotomy) vs. orthosis vs. watchful waiting (control) | | Yes | Yes - Pain during walking (VAS) | | Supportive of operative procedure | (Surgery vs. orthosis and surgery vs. control)   1. Subjective improvement - Y 2. Number of painful days - Y 3. Cosmetic disturbance - Y 4. Footwear problems - Y 5. Functional status (AOFAS) - Y 6. Satisfaction -Y |
|  | Conclusion: Surgical osteotomy is an effective treatment for painful hallux valgus. Orthoses provide short-term symptomatic relief. | | | | | | | | | |
| **Open Reduction of Shoulder Dislocation** | | | | | | | | | | |
| 1. Bottoni 2002 | 24 adult males with primary anterior dislocation of the shoulder | | | Arthroscopic stabilisation + rehabilitation vs. sling-immobilisation + rehabilitation | | No | No | | NA | 1. Failure – Y 2. Functional – Y 3. Patient satisfaction – Y |
|  | Conclusion: Arthroscopic stabilization of traumatic, first-time anterior shoulder dislocations is an effective and safe treatment that significantly reduces the recurrence rate of shoulder dislocations in young athletes when compared with conventional, non-operative treatment. | | | | | | | | | |
| 2. Edmonds 2003 | 24 skeletally mature patients with primary traumatic anterior dislocation of the shoulder | | | Sling immobilisation + rehabilitation vs. arthroscopic stabilisation + rehabilitation | | Yes | Yes – Proprioception (threshold to detection of passive motion (TTDPM) and reproduction of passive positioning (RPP) | | Not supportive of operative procedure | Nil |
|  | Conclusion: These findings suggest that treatment by early arthroscopic stabilization and rehabilitation after primary traumatic anterior dislocation of the shoulder does not enhance proprioception more than standard immobilization and re- habilitation. | | | | | | | | | |
| 3. Jakobsen 2007 | 76 skeletally mature patients with first-time traumatic anterior shoulder dislocation | | | Open repair + rehabilitation vs. sling immobilisation + rehabilitation | | No | Yes – Recurrence | | Supportive of operative procedure | 1. Oxford Score – Y |
|  | Conclusion: Because open repair produces superior results compared with conservative treatment, we recommend that the surgeon consider performing primary repair in active patients to reduce the risk of recurrence | | | | | | | | | |
| 4. Kirkley 1999 | 40 skeletally mature patients with first traumatic anterior dislocation of the shoulder | | | Immobilisation for 3 weeks + rehabilitation vs. arthroscopic stabilisation + immobilisation for 3 weeks + rehabilitation | | Yes | Yes – redislocation | | Supportive of operative procedure | 1. QOL (Western Ontario Shoulder Instability Index) – Y |
|  | Conclusion: Arthroscopic stabilization significantly reduces the rate of redislocation in patients younger than 30 years of age who have sustained a first traumatic anterior dislocation of the shoulder without negatively affecting range of motion. More importantly, the 2-year follow-up of these patients suggests that arthroscopic stabilization may afford better disease-specific quality of life than taking a wait-and-see approach. | | | | | | | | | |
| 5. Wintzell 1999 | 60 skeletally mature patients with traumatic primary anterior shoulder dislocation | | | Arthroscopic lavage + rehabilitation vs. non-operative treatment with optional sling for 1 week then free mobilisation + rehabilitation | | No | No | | NA | 1. Recurrence – Y 2. Stability – Y 3. Functional – Y 4. Return to previous activity – Y |
|  | Conclusion: We conclude that arthroscopic lavage reduced the recurrence rate and produced a better functional outcome at 1-year follow-up than the non-operative treatment in young individuals. | | | | | | | | | |
| 6. Wintzell 2000 | 16 adults with traumatic primary anterior shoulder dislocation | | | Arthroscopic lavage + rehabilitation vs. non-operative treatment with optional sling for 1 week then free mobilisation + rehabilitation | | No | Yes - Joint effusion | | Supportive of operative procedure | Nil |
|  | Conclusion: The joint effusion decreased more rapidly (33%) in the group treated with arthroscopic lavage (P = 0.02) than in the non-operated group. | | | | | | | | | |
| **Open Reduction of Acromioclavicular Dislocation** | | | | | | | | | | |
| 1. Bannister 1989 | 60 adults with acute acromioclavicular dislocation | | | Broad-arm sling + rehabilitation vs. open reduction and fixation with coracoclavicular screw + rehabilitation | | No | No | | NA | 1. Functional (imatani scoring system)– N 2. Time-to-return to work – N |
|  | Conclusion: Our results indicate that in the average case, the closed treatment of acute acromioclavicular dislocation is superior to early open reduction and coracoclavicular screw fixation. | | | | | | | | | |
| 2. Imatani 1975 | 23 skeletally mature patients with acute, complete acromioclavicular separation. | | | Open reduction and internal fixation vs. sling immobilisation | | No | No | | NA | 1. Functional (Imatani scoring system) - N 2. Radiological – N |
|  | Conclusion: Minimum immobilization and early rehabilitation of the shoulder was the recommended treatment of choice. | | | | | | | | | |
| 3. Larsen 1986 | 84 adults with total acromioclavicular dislocation | | | Open reduction and internal fixation + sling immobilisation + rehabilitation vs. sling immobilisation + rehabilitation | | No | No | | NA | 1. Clinical – N 2. Global – N |
|  | Conclusion: For most patients with total acromioclavicular dislocation we recommend conservative treatment with a sling until the patient is free of pain. Operation should be considered in thin patients who have a prominent lateral end of the clavicle, in those who do heavy work and in patients whose daily work requires that the shoulder often be held about 90 degrees of abduction and flexion. | | | | | | | | | |
| **Open Reduction of Patella Dislocation** | | | | | | | | | | |
| 1. Camanho 2009 | 33 patients (12 to 74 years) with first episode of patellofemoral dislocation | | | Mediopatellofemoral ligament repair vs. immobilisation | | No | No | | NA | 1. Recurrence – Y 2. Functional (Kujala score) – Y |
|  | Conclusion: We conclude that surgical treatment afforded better results. There were no recurrences in the surgical treatment group, but there were 8 recurrences in the conservative treatment group. The mean Kujala score was 92 in the surgical treatment group and 69 in the conservative treatment group. | | | | | | | | | |
| 2. Christiansen 2008 | 80 patients (13-30 years) with first time dislocation of the patella | | | Mediopatellofemoral ligament repair vs. knee brace with 0-20 degree ROM | | Yes | Yes –   1. Redislocation 2. Functional (Kujala score) | | Not supportive of operative procedure | Nil |
|  | Conclusion: Delayed primary repair of the MPFL by use of an anchor-based reattachment to the adductor tubercle without vastus medialis obliquus repair after primary patella dislocation does not reduce the risk of redislocation nor does it produce any significantly better subjective functional outcome based on the Kujala knee score. | | | | | | | | | |
| 3. Palmu 2008 | 71 patients (<16 years) with acute patella dislocations without large intra-articular fragments | | | Operative treatment (repair of all medial structures +/- lateral release) vs. non-operative treatment | | No | Yes - Recurrence | | Not supportive of operative procedure |  |
|  | Conclusion: Initial operative repair of the medial structures combined with lateral release did not improve the long-term out- come, despite the very high rate of recurrent instability. | | | | | | | | | |
| 4. Nikku 1997 | 125 patients (9-47 years) with primary dislocation of the patella | | | Surgical repair vs. immobilisation | | No | No | | NA | 1. Subjective global assessment – N 2. Recurrence – N 3. Clinical – N |
|  | Conclusion: The recurrence of patellar dislocation may be more frequent than reported, whatever the form of treatment. Routine operative management cannot be recommended for primary dislocation of the patella. | | | | | | | | | |
| **Knee, Repair of Cruciate Ligament** | | | | | | | | | | |
| 1. Andersson 1992 | 107 patients with acute ACL rupture | | | ACL repair (with or without collateral ligament repair) + rehabilitation vs. rehabilitation only | | No | No | | NA | 1. Knee Laxity – Y 2. Knee performance - equivocal 3. Muscle torque – N 4. Return to normal activity – N 5. Subjective global assessment - Y |
|  | Conclusion: Augmented repair of the ACL resulted in superior stability with better subjective knee function at higher levels of activity than results achieved with nonsurgical treatment of isolated ACL injuries | | | | | | | | | |
| 2. Odensten 1983 | 90 patients (13 to 49 years) with total mid-structural tears of the anterior cruciate ligament | | | Repair (+/- augmentation) vs. non-operative | | No | No | | NA | 1. Clinical – N 2. Functional – N 3. Stability - Y 4. Return to normal activity – N |
|  | Conclusion: The present study suggests that early primary suture with augmentation may give the patient with an acutely torn ACL a better start than conservative treatment, although conservative treatment is sometimes followed by a good primary result. A long-term follow- up study is needed to determine criteria for the selection of patients for operation or conservative treatment. | | | | | | | | | |
| 3. Sandberg 1987 | 200 patients (15-61 years) with injury to either the ACL or MCL or both | | | Suture repair of ligament/s without augmentation vs. immobilisation in cast | | No | No | | NA | 1. Clinical - equivocal 2. Functional – N 3. Duration of disability – N |
|  | Conclusion: With injuries to the anterior cruciate ligament, recovery was more rapid without surgery but otherwise the results differed between the two groups in only one respect: the pivot-shift test was more often positive after conservative treatment. The results were good in both treatment groups even though most of the patients who had an injury of the anterior cruciate ligament were somewhat less pleased with the outcome after a period of time. | | | | | | | | | |
| **Repair of Achilles Tendon Rupture** | | | | | | | | | | |
| 1. Nilsson-Helander 2010 | 97 patients (16-65 years) with acute Achilles tendon rupture | | | Surgical repair + rehabilitation vs. immobilisation + rehabilitation | | Yes | Yes – rerupture rate | | Not supportive of operative procedure | 1. Patient reported outcome and physical activity – N 2. Clinical – N |
|  | Conclusion: The results of this study did not demonstrate any statistically significant difference between surgical and nonsurgical treatment. | | | | | | | | | |
| 2. Cetti 1993 | 111 adults with acute rupture of the Achilles tendon | | | Surgical repair + immobilisation in cast vs. immobilisation in cast | | No | No | | NA | 1. Self-reported symptoms – Y 2. Clinical – Y 3. Functional – N 4. Resumption of sporting activities - Y 5. Complications – N |
|  | Conclusion: The conclusion we reached through this randomized prospective study is that operative treatment of reiptured Achilles tendons is preferable but nonoperative treatment is an acceptable alternative. | | | | | | | | | |
| 3. Majewski 2000 | 73 patients with Achilles tendon ruptures | | | Operative “end-to-end surgery” vs. percutaneous repair vs. conservative therapy | | No | No | | NA | 1. Achilles tendon score – N 2. Functional ability - N |
|  | Conclusion: Bearing in mind the literature and these results, we have developed an algorithm for treating Achilles tendon rupture to assist decision making in daily routine. In this way, the use of percutaneous Achilles tendon repair can be carried out in most of cases. | | | | | | | | | |
| 4. Metz 2008 | 83 patients with acute Achilles tendon rupture | | | Minimally invasive surgical treatment vs. nonoperative treatment by functional bracing | | Yes | Yes - All complications except rerupture | | Not supportive of operative procedure | 1. Time to work resumption - Y 2. Participation in sports - N 3. Patient satisfaction - N 4. Pain - N |
|  | Conclusion: There appears to be a clinically important difference in the risk of complications between minimally invasive surgical treatment and nonoperative treatment for acute Achilles tendon ruptures, but this was not statistically significant. | | | | | | | | | |
| 5. Moller 2001 | 112 patients (16-65 years) with acute Achilles tendon rupture | | | Surgical repair (end-to-end suture) + rehabilitation vs. immobilisation in below knee cast + rehabilitation | | No | No | | NA | 1. Rerupture – Y 2. Clinical – N 3. Time to return to activities - N |
|  | Conclusion: The rate of rerupture was 20.8% after non-surgical and 1.7% after surgical treatment (p < 0.001). Surgical and non-surgical treatment produced equally good functional results if complications were avoided. However, the rate of rerupture after non-surgical treatment was unacceptably high. | | | | | | | | | |
| 6. Nistor 1981 | 105 consecutive adult patients with closed acute Achilles tendon rupture | | | End-to-end surgical repair + below knee cast vs. immobilisation in below knee cast | | No | No | | NA | 1. Clinical – N 2. Functional - N 3. Subjective – N |
|  | Conclusion: I concluded that non-surgical treatment offers advantages over surgical treatment. | | | | | | | | | |
| 7. Thermann 1995 | 50 patients with Achilles tendon rupture | | | Operative treatment vs. conservative (immobilisation in a newly developed boot) | | No | No | | NA | 1. Functional – N 2. Course of healing – N |
|  | Conclusion: In this trial there were no significant differences either in the functional results or in the course of healing. | | | | | | | | | |
| 8. Twaddle 2007 | 42 patients (18-50 years) with acute rupture of the Achilles tendon | | | Surgical (end-to-end suture) vs. conservative (immobilisation in a hanging equinus POP) | | Yes | Yes - Re-rupture | | Not supportive of operative procedure | 1. Clinical – N |
|  | Conclusion: There appears to be no difference between the 2 groups, suggesting that controlled early motion is the important part of treatment of ruptured Achilles tendon. | | | | | | | | | |
| 9. Willitis 2010 | 144 patients with acute Achilles tendon rupture | | | Operative (end-to-end suture) _ accelerated functional rehabilitation program vs. non-operative (immobilisation + accelerated functional rehabilitation program) | | Yes | Yes - Re-rupture | | Not supportive of operative procedure | 1. Clinical – N |
|  | Conclusion: This study supports accelerated functional rehabilitation and nonoperative treatment for acute Achilles tendon ruptures. | | | | | | | | | |
| **Humerus, Distal, Treatment of Fracture by Open Reduction with Internal Fixation** | | | | | | | | | | |
| 1. Pandey 2008 | 60 paediatric patients with with closed extension type Gartland type IIB and III supracondylar fracture of the humerus | | | Closed reduction + immobilisation vs. closed reduction +crossed K-wire fixation | | No | No | | NA | 1. Radiological – N 2. Functional – N |
|  | Conclusion: The outcome of displaced extension type supracondylar fracture of the humerus in children, managed with closed reduction and slab application are comparable with closed reduction and crossed Kirschner wire fixation in terms of range of motion but is inferior in restoration of carrying angle. Good to excellent cosmetic and functional results are higher with crossed percutaneous Kirschner wires fixation than with slab immobilization. | | | | | | | | | |
| **Joint arthrodesis** | | | | | | | | | | |
| 1. Wood 2003 | 47 patients with stable thoracolumbar burst fractures with no neurological deficits | | | Operative (posterior or anterior arthrodesis and instrumentation) vs. nonoperative (application of body cast or orthosis) | | No | No | | NA | 1. Radiological – N 2. Pain - N 3. Disability (Functional) – Equivocal |
|  | Conclusion: We found that operative treatment of patients with a stable thoracolumbar burst fracture and normal findings on the neurological examination provided no major long-term advantage compared with nonoperative treatment. | | | | | | | | | |
| **Abscess Drainage** | | | | | | | | | | |
| 1. Ernst 1995 | 27 adults with fluctuant inguinal buboes | | | Needle aspiration vs. incision and drainage + packing | | No | Yes – Retreatment | | Supportive of operative procedure | Nil |
|  | Conclusion: Incision and drainage is an effective method of treating fluctuant buboes and may be preferable to traditional needle aspiration considering the frequency of re-aspirations in the study patients. Limitations of this study include lack of complete laboratory testing and lack of follow-up of all patients. | | | | | | | | | |
| 2. Eryilmaz 2005 | 55 lactating women with breast abscesses | | | Incision and drainage vs. 2^nd^ daily needle aspirations | | No | No | | NA | 1. Failure rate – Y 2. Mean healing time – N |
|  | Conclusion: Breast abscesses smaller than 5 cm in diameter on physical examination can be treated with repeated aspirations with good cosmetic results. Incision and drainage should be reserved for use in patients with larger abscesses. | | | | | | | | | |
| 3. Singh 1989 | 50 patients (21 to 72 years) with resistant amoebic liver abscesses | | | IM dehydroemetine (conservative) vs. US-guided needle aspiration vs. US-guided catheter drainage vs. ope surgical drainage with catheter insertion | | No | No | | NA | 1. Complete clinical relief – N 2. Failure – N 3. Morbidity – N 4. Sonographic resolution of cavities - Y |
|  | Conclusion: The most impressive results were seen with percutaneous catheter drainage. This new modality of treatment is recommended for all resistant cases of amoebic liver abscesses. | | | | | | | | | |
| 4. Spires 1987 | 62 patients (12 to 53 years) with peritonsillar abscesses | | | Needle aspiration vs. transoral incision and drainage | | No | No | | NA | 1. Failure – N 2. Average recovery time – N |
|  | Conclusion: the advantages if needle aspiration alone as the primary drainage procedure outweigh the acceptably low failure rate, amking it the initial procedure of choice. | | | | | | | | | |
| 5. Stringer 1988 | 52 patients (13 to 60 years) with aspiration-prove peritonsillar abscess | | | Needle aspiration vs. incision and drainage | | No | Yes – Failure | | Not supportive of operative procedure | Nil |
|  | Conclusion: These data indicate that outpatient permucosal needle drainage of peritonsillar abscess is an acceptable, inexpensive treatment comparing favorably with incision and drainage. | | | | | | | | | |
| **Clavicle, Treatment of Fracture, Open Reduction with Internal Fixation** | | | | | | | | | | |
| 1. Judd 2009 | 57 patients (17-40 years) with isolated acute, displaced, or angulated, closed fractures of the middle third of the clavicle | | | Immediate operative stabilisation with a modified Hagie pin vs. nonoperative therapy (sling + motion as tolerated + activity restrictions) | |  | No | | NA | 1. Radiological – N 2. Complications – N 3. Functional – N |
|  | Conclusion: Results of this study suggest that, though patients with midshaft clavicle fractures had higher functional scores at short-term follow-up after internal fixation, functional scores were similar at 6 months and 1 year. In addition, internal fixation with a modified Hagie pin was associated with a higher complication rate. | | | | | | | | | |
| 2. Altamimi 2008 | 132 patients (16 to 60 years) with displaced midshaft fractures of the clavicle | | | Operative (plate-fixation) vs. nonoperative (sling) | | Yes | Yes - Shoulder function scores (Constant Shoulder Score+ DASH) | | Supportive of operative procedure | 1. Radiological – Y 2. Complications – Y 3. Satisfaction - Y |
|  | Conclusion: Operative fixation of a displaced fracture of the clavicular shaft results in improved functional outcome and a lower rate of malunion and nonunion compared with nonoperative treatment at one year of follow- up. | | | | | | | | | |
| 3. Smekal 2009 | 60 patients (18-65) with fully displaced midshaft clavicular fractures | | | Operative (elastic stable intramedullary nailing) vs. non-operative (sling for comfort and mobilization as tolerated) | | No | No | | NA | 1. Complications - Y 2. Disability (DASH score) – N 3. Global (Constant Shoulder score) – Y 4. Clavicular shortening – Y 5. Satisfaction – N |
|  | Conclusion: ESIN of displaced midshaft clavicular fractures resulted in a lower rate of nonunion and delayed union, a faster return  to daily activities, and a better functional outcome. Clavicular shortening was significantly lower, and overall satisfaction was higher in the operative group. | | | | | | | | | |
| **Humerus, Proximal, Treatment of Fracture, Open Reduction with Internal Fixation** | | | | | | | | | | |
| 1. Fjalestad 2010 | 50 patients (60 years or over) with severely displaced three- or four-part fractures of the proximal humerus | | | Surgical (angular stable interlocking implant) vs. conservative (immobilised in a modified Velpeau bandage) | | Yes | Yes - Quality of life years and societal costs (resource use) | | Not supportive of operative procedure |  |
|  | Conclusion: There was no significant difference in QALYs or costs between surgical and conservative treatment of severe displaced proximal humeral fractures. | | | | | | | | | |
| 2. Zyto 1997 | 40 patients (mean age 74 years) with displaced three- or four-part fractures of the humerus | | | Conservative (supportive sling + physiotherapy) vs. surgical (tension-band osteosynthesis) | | No | No | | NA | 1. Functional – N 2. Pain – N 3. Complications – N 4. Radiological – N |
|  | Conclusion: Semi-rigid fixation with tension-band wiring of displaced multifragment fractures of the proximal humerus in the elderly did not improve the functional outcome when compared with conservative treatment. | | | | | | | | | |
| **Release of Carpal Tunnel** | | | | | | | | | | |
| 1. Garland 1964 | 22 women (35 to 63 years) with clinical and electromyographic evidence of carpal tunnel syndrome (CTS) | | | Operative (open release of anterior carpal ligament) vs. conservative (POP wrist-splint for 1 month) | | No | No | | NA | 1. Clinical – Y 2. Electromyographical - Y |
|  | Conclusion: Altogether twenty-even operations were performed and in all of them the electromyographic finding returned to normal. Twenty-four were clinically cured a year later and three still complained of mild parasthesia without significant distress. | | | | | | | | | |
| 2. Gerritsen 2002 | 176 adults with clinically and electrophysiologically confirmed CTS | | | Conservative (nocturnal wrist splinting for 6 weeks) vs. operative (open carpal tunnel release) | | Yes | Yes - success rates | | Supportive of operative procedure | 1. No. of nights waking due to symptoms – N 2. Severity of main complaint - Y 3. Symptom severity score (subjective) – Y 4. Functional status score – Y 5. Overall severity of complaint (objective) - Y |
|  | Conclusion: Treatment of open carpal tunnel release surgery resulted in better outcomes than treatment with wrist splinting for patients with CTS. | | | | | | | | | |
| 3. Jarvik 2009 | 116 patients with electrodiagnostic evidence of CTS | | | Carpal tunnel surgery vs. structured non-surgical treatment (hand therapy + ultrasound) | | Yes | Yes – Functional status) Carpal Tunnel Syndrome Assessment Questionnaire – CTSAQ) | | Supportive of operative procedure | Subcomponents of CTSAQ   1. Severity of symptoms – Y 2. Effect on work and other activities – N |
|  | Conclusion: Symptoms in both groups improved, but surgical treatment led to better outcome than did non-surgical treatment. However, the clinical relevance of this difference was modest. Overall, our study confirms that surgery is useful for patients with carpal tunnel syndrome without denervation. | | | | | | | | | |
| 4. Korthals-de Bos 2006 | 176 patients (>18 years) with clinical and electrophysiologically confirmed CTS | | | Conservative (nocturnal splinting for 6 weeks) vs. open carpal tunnel release surgery | | No | Yes – Cost effectiveness | | Supportive of operative procedure | 1. Costs (direct & indirect) – N 2. Clinical - Equivocal |
|  | Conclusion: In the Netherlands, surgery is more cost-effective compared with splinting, and recommended as the preferred method of treatment for patients with CTS. | | | | | | | | | |
| 5. Ly-Pen 2005 | 101 patients (>18 years) or 163 wrists with clinical and neurophysiological diagnosis of CTS | | | Surgical (limited palmar incision technique) vs. local steroid injection | | Yes | Yes – Improvement in nocturnal paraesthesia (defined by >=20% improvement in AS scores) | | Not supportive of operative procedure | 1. Diurnal pain – N 2. Functional impairment – N |
|  | Conclusion: Over the short term, local steroid injection is better than surgical decompression for the symptomatic relief of CTS. At 1 year, local steroid injection is as effective as surgical decompression for the symptomatic relief of CTS. | | | | | | | | | |

* If statistical significance was not stated, the difference was determined to be not statistically significant
