# Supplementary material for: Randomised Trial Support for Orthopaedic Surgical Procedures
Source: PLoS One. 2014 Jun 13;9(6):e96745. doi: 10.1371/journal.pone.0096745 (PMC4057075; doi:10.1371/journal.pone.0096745)
Supplement: Appendix S5 — Risk of Bias assessment of 83 included RCTs using the Cochrane Collaboration's Risk of Bias tool. (PDF) [file pone.0096745.s005.pdf]

APPENDIX 5

Risk of bias assessment of 83 included RCTs using the Cochrane Collaboration's Risk of Bias tool.

	Random sequence generation (selection bias)	Allocation concealment (selection bias)	Blinding of participants and personnel (performance bias)	Blinding of outcome assessment (detection bias)	Incomplete outcome data (attrition bias)	Selective reporting (reporting bias)
Abdel-salam 1991	+	?	-	?	-	+
Altamimi 2008	+	+	-	?	-	+
Andersson 1992	-	-	?	?	+	+
Arden 2008	+	+	-	+	-	+
Azzopardi 2005	+	?	-	-	?	+
Bannister 1989	+	?	-	-	+	+
Bauer 1985	+	?	-	-	+	+
Bong 1981	?	?	-	-	?	+
Bottoni 2002	-	-	-	-	+	-
Brox 1993	+	?	-	+	?	+
Buckley 2002	+	+	-	?	+	+
Camanho 2009	+	+	-	-	+	+
Cetti 1993	?	?	-	-	+	+
Chang 1993	+	+	-	+	-	+
Christiansen 2008	+	+	-	?	+	+
Edmonds 2003	?	?	-	+	+	+
Ekenstam 1989	-	-	-	?	?	+
Ernst 1995	+	?	-	-	+	?
Eryilmaz 2005	?	?	-	-	+	+
Fjalestad 2010	+	+	-	?	+	+
Foster 2003	?	+	-	-	+	+
Fulford 1993	-	-	-	?	+	+
Garland 1964	?	?	-	?	+	+
Gerristen 2002	+	+	-	-	+	+
Gupta 1999	?	?	-	-	+	+
Haahr 2005	+	+	-	-	+	+
Herrlin 2006	?	?	-	+	+	+
Hooper 1991	+	?	-	-	+	+
Hornby 1989	+	+	-	+	-	+
Imatani 1975	-	-	-	-	?	+
Jakobsen 2007	+	+	-	-	+	-
Jarvik 2009	+	+	-	-	+	+
Judd 2009	+	+	-	-	+	+
Kalunian 2000	+	?	+	+	+	+
Kapoor 2000	?	?	-	-	?	+
Karladani 2000	?	?	-	-	-	+
Ketola 2009	+	+	-	+	+	+
Kettunen 2007	+	+	-	+	+	+
Kirkley 1999	-	-	-	+	+	+
Kirkley 2008	+	+	-	+	+	+
Korthals-de Bos 2006	?	?	-	-	+	+
Larsen 1986	+	+	-	+	+	+
Livesley 1991	-	-	-	-	+	+
Ly-Pen 2005	+	+	-	-	+	+
Majewski 2000	?	?	-	?	?	?
Makwana 2001	+	-	-	-	-	+
McLauchlan 2002	+	+	-	-	+	+
McQueen 1996	?	+	-	?	?	+
Metz 2008	+	+	-	-	+	+
Moller 2001	+	+	-	-	+	+
Moonsmayer 2010	+	+	-	+	-	+
Moseley 2002	+	+	+	+	+	+
Nikku 1997	-	-	-	-	+	+
Nilsson-Helander 2010	+	+	-	-	+	+
Nistor 1981	-	-	-	-	+	+
Odensten 1983	?	?	?	?	?	+
Palmu 2008	-	-	-	-	+	+
Pandey 2008	?	?	-	-	+	+
Parmar 1993	?	?	-	?	-	+
Peters 1997	?	?	+	?	?	?
Phillips 1985	+	+	-	?	-	+
Rodriguez-Merchan 1997	+	+	-	-	?	+
Rowley 1986	-	-	-	?	+	?
Salai 2000	-	-	-	-	-	+
Sandberg 1987	+	+	-	-	+	+
Shankar 1992	?	?	-	?	+	+
Singh 1989	?	?	-	-	+	+
Smekal 2009	+	+	-	-	+	+
Spires 1987	-	-	?	?	+	+
Stoffelen 1998	?	?	-	?	+	+
Stringer 1988	?	?	-	-	+	+
Thermann 1995	?	?	-	?	?	?
Torkki 2001	+	+	-	-	+	+
Twaddle 2007	+	+	-	?	+	+
Van der Linden 1979	?	?	+	+	+	+
Willitis 2010	+	+	-	?	+	+
Wintzell 1999	+	+	-	+	+	+
Wintzell 2000	+	+	-	+	+	+
Wong 2010	?	+	-	+	+	+
Wood 2003	+	?	-	?	+	+
Xu 2008	?	?	?	?	?	?
Zyluk 2007	?	?	?	?	-	+
Zyto 1997	+	+	-	-	-	+
